# Amination of ω-Functionalized Aliphatic Primary Alcohols by a Biocatalytic Oxidation–Transamination Cascade

**DOI:** 10.1002/cctc.201500589

**Published:** 2015-09-03

**Authors:** Mathias Pickl, Michael Fuchs, Silvia M Glueck, Kurt Faber

**Affiliations:** a Austrian Centre of Industrial Biotechnology (ACIB GmbH)Petersgasse 14, 8010, Graz (Austria); b Department of Chemistry, Organic&Bioorganic Chemistry, University of GrazHeinrichstrasse 28, 8010, Graz (Austria) Fax: (+43) 316-380-9840 E-mail: Kurt.Faber@Uni-Graz.at

**Keywords:** alcohol oxidase, amination, biocatalysis, cascade, transaminase

## Abstract

Amination of non-activated aliphatic fatty alcohols to the corresponding primary amines was achieved through a five-enzyme cascade reaction by coupling a long-chain alcohol oxidase from *Aspergillus fumigatus* (LCAO_Af) with a ω-transaminase from *Chromobacterium violaceum* (ω-TA_Cv). The alcohol was oxidized at the expense of molecular oxygen to yield the corresponding aldehyde, which was subsequently aminated by the PLP-dependent ω-TA to yield the final primary amine product. The overall cascade was optimized with respect to pH, O_2_ pressure, substrate concentration, decomposition of H_2_O_2_ (derived from alcohol oxidation), NADH regeneration, and biocatalyst ratio. The substrate scope of this concept was investigated under optimized conditions by using terminally functionalized C_4_–C_11_ fatty primary alcohols bearing halogen, alkyne, amino, hydroxy, thiol, and nitrile groups.

Biocatalytic cascades have emerged as a time-, resource-, and cost-saving strategy in bioorganic synthesis.^1^ The use of several enzymes in a one-pot fashion avoids purification/isolation of (unstable) intermediates and the associated unavoidable loss of material. Numerous examples of multienzymatic processes of ever increasing complexity for the production of valuable compounds^2^ indicate that the areas of “systems biocatalysis”^3^ and pathway engineering^4^ are beginning to merge. The synthesis of amines dominates current cascade design because the occurrence of amines is underrepresented in the pool of renewable carbon sources, in contrast to their frequent need in chemical synthesis.^5^

For instance, terminal alkylamino functionalization of alkanes and fatty acid methyl esters was achieved by combining an alkane monooxygenase (AlkBGT) and a ω-transaminase in a single designed whole-cell system.^6^ The coupling of a ω-transaminases with other enzymes, such as acetohydroxyacid synthase, transketolase, various hydrolases, and alcohol dehydrogenases enabled the synthesis of (chiral) amine derivatives.^7^

The direct transformation of alcohols to amines is only feasible by metal catalysts,^8^ no enzyme is known for this reaction. However, biocatalytic two-step oxidation–reductive amination sequences are known. Oxidation of an alcohol by an alcohol dehydrogenase yields the corresponding aldehyde/ketone, which can be reductively aminated by an ω-transaminase. The elegance of this redox-neutral process is the internal cofactor recycling, in which NADH generated during alcohol oxidation is employed in the reductive amination step. This concept has been successfully applied to a broad range of linear and cyclic aliphatic primary and secondary alcohols, aryl-alkanols, benzylic alcohols, and α,ω-diols for the synthesis of (di)amines.^7^

Alcohol oxidases represent an attractive, but underrepresented, alternative to (thermodynamically disfavoured) nicotinamide-dependent alcohol oxidation catalyzed by alcohol dehydrogenases. These enzymes are commonly flavin- or Cu-dependent and use O_2_ as an electron acceptor.^9^ Two-electron transfer yields H_2_O_2_ as a byproduct, which is destroyed by catalase or by the horseradish peroxidase (HRP)/2,2′-azino-bis(3-ethylbenzothiazoline-6-sulfonic acid (ABTS) system. This method is a “green” alternative to traditional protocols, which require transition metals, dimethylsulfoxide (e.g. Swern, Pfitzner–Moffat oxidation),^10^,^11^ or nitroxyl radicals (e.g. TEMPO).^12^

Recently, we established a two-step one-pot oxidation–transamination cascade based on Cu^I^-dependent galactose oxidase (GOase) in combination with a ω-transaminase.^13^ Dictated by the substrate characteristics of GOase from *Fusarium* NRRL 2903, only electronically activated benzylic and cinnamic alcohols were accepted, and this method was not applicable to nonactivated aliphatic (fatty) alcohols. To broaden the substrate scope of this protocol, a search for a suitable alcohol oxidase revealed a putative flavin-dependent long-chain alcohol oxidase from *Aspergillus fumigatus* (LCAO_Af) as a promising candidate.^14^ The enzyme shows homology and sequence identity (30–40 %) with other flavoprotein alcohol oxidases and contains the conserved flavin-binding domain (pfam00732) of the glucose–methanol–choline (GMC) oxidase family. LCAO shows activity in the H_2_O_2_/HRP/ABTS and supplementary flavin adenine dinucleotide (FAD) enhances the activity of the enzyme (data not shown). Preliminary results reported the oxidation of C_6_–C_8_ fatty alcohols yielded the corresponding aldehydes. Reductive amination of aliphatic aldehydes through ω-transaminases is well known.^15^ To shift the unfavorable equilibrium towards the amine, the well-established l-alanine donor system, employing an alanine dehydrogenase (Ala-DH) combined with an adequate NADH-recycling system, completed the overall cascade (Scheme 1).^16^ In contrast to Cu^I^-dependent GOase, where alcohol oxidation selectively stops at the aldehyde stage, undesired over oxidation of aldehydes to carboxylic acids (a common phenomenon for flavin-dependent alcohol oxidases) by LCAO_Af had to be taken into account.^17^

**Scheme 1:**
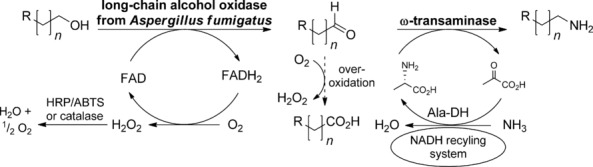
Biocatalytic oxidation–transamination of ω-functionalized nonactivated fatty alcohols.

The overall performance of the cascade was tuned with respect to the following parameters by using 1-hexanol as substrate (detailed data are given in the Supporting Information):

1. For the removal of H_2_O_2_, disproportionation catalyzed by catalase from *Micrococcus lysodeiktikus* or two-electron transfer mediated by HRP employing ABTS as an electron sink worked equally well. The latter (chromophoric) approach has advantages in screening conditions, whereas the former is more suitable for preparative-scale transformations (Supporting Information, Table S1).
2. For the reductive amination step, various nicotinamide cofactor recycling systems based on glucose/glucose dehydrogenase (GDH), formate/formate dehydrogenase (FDH), and phosphite/phosphite dehydrogenase (PtDH) were compared: The phosphite/PtDH system was the least efficient, whereas the formate/FDH system led to significant over oxidation of the aldehyde intermediate to yield the undesired carboxylic acid. The best results were obtained with glucose/GDH by using standard conditions, but this result was not further optimized (Table S2).
3. A significant advantage of separate overexpression of the oxidase and the ω-transaminase is the possibility to adjust the ratio of both enzymes. The best results were obtained when LCAO Af and ω-TA Cv (employed as whole lyophilized cells) were used in a ratio of 2:1 (Table S3).
4. The pH profile was investigated within a range of pH 5–12 (Figure S2), which revealed that the efficiency of the cascade continuously increased from pH 6 (conversion <2 %) to a maximum at pH 10, followed by a sharp drop at pH 12.
5. The efficiency of the cascade was very sensitive towards elevated product concentrations, deduced from the fact that increasing amounts of substrate (10–75 mm) led to a steady decrease in conversion and gave comparable levels of product (≈18–20 mm, Figure S3).
6. Since the solubility of the oxidant O_2_ in aqueous systems is limited, experiments were conducted under atmospheric conditions and at elevated pressure, which revealed an optimal performance at 2–4 bar (Table S4).
7. To evaluate the overall performance of the optimized cascade reaction compared to the initial conditions, the amine formation was followed over time. Engineering of the reaction parameters led to a considerable improvement of the overall efficiency with full conversion of 1-hexanol to the corresponding amine within 10 h (Figure S4).

To evaluate the substrate tolerance (see Table 1) of the biocatalytic oxidation–transamination cascade reaction, primary alcohols with a chain length ranging from C_4_–C_11_ and derivatives bearing a halogen, alkyne, amino, hydroxy, thiol, and nitrile group were subjected to the enzymatic amination cascade.

**Table 1:** Substrate scope of the biocatalytic oxidation–transamination cascade.
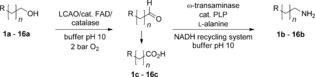

Entry	Substrate			Product		Byproduct	
		*n*	R		[%]^[a]^		[%]
1	**1 a**	3	H	**1 b**	20	**1 c**	<1
2	**2 a**	4	H	**2 b**	75	**2 c**	<1
3	**3 a**^[b]^	5	H	**3 b**	>99	**3 c**	<1
4	**4 a**	6	H	**4 b**	>99	**4 c**	<1
5	**5 a**	7	H	**5 b**	>99	**5 c**	<1
6	**6 a**	8	H	**6 b**	82	**6 c**	5^[c]^
7	**7 a**	9	H	**7 b**	46	**7 c**	20^[c]^
8	**8 a**	10	H	**8 b**	16	**8 c**	26^[c]^
9	**9 a**	5	Br	**9 b**	74	**9 c**	<1
10	**10 a**	5	Cl	**10 b**	98	**10 c**	<1
11	**11 a**	7	Cl	**11 b**	96	**11 c**	<1
12	**12 a**	5	C≡N	**12 b**	42	**12 c**	<1
13	**13 a**	5	C≡CH	**13 b**	>99	**13 c**	<1
14	**14 a**	5	NH_2_	**14 b**	n.c.	**14 c**	n.c.
15	**15 a**	5	OH	**15 b**	n.c.	**15 c**	n.c.
16	**16 a**	5	SH	**16 b**	n.c.	**16 c**	n.c.

Reaction conditions: Sodium phosphate buffer (100 mm, pH 10), substrate (10 mm), l-alanine (100 mm), glucose (80 mm), NAD^+^ (2 mm), NH_4_Cl (67 mm), PLP (2 mm), FAD (1 mm), LCAO_Af (40 mg whole lyophilized *E. coli* cells), ω-TA_Cv (20 mg whole lyophilized *E. coli* cells), GDH (2 U), Ala-DH (0.013 U), catalase (1700 U), 20 h at RT, 170 rpm and 2 bar O_2_; n.c.=no conversion, [a] conversion refers to the formed amine as determined by GC-MS analysis after derivatization; [b] used in optimization studies; [c] owing to peak tailing, the accuracy for the determination of the corresponding carboxylic acid was ±5 %.

In the first screening, various unfunctionalized linear aliphatic alcohols with a chain length ranging from four to eleven carbon atoms were tested, and the performance was measured as overall conversion to the corresponding amine **1 b**–**8 b** (Table 1, entries 1–8). Although short 1-butanol (**1 a**) was converted only moderately (conv.=20 %), significantly enhanced amine formation was observed by increasing the chain length, leading to quantitative conversions for C_6_–C_8_ alcohols (**3 a**–**5 a**, entries 3–5). This effect nicely correlates with the fact that a “long-chain” alcohol oxidase was used in the oxidation step. Beyond this range, conversions gradually decreased again and reached only 16 % for 1-undecanol (**8 a**). The decrease in amine formation for long-chain alcohols was accompanied by significant over oxidation to the corresponding carboxylic acid (**6 c**–**8 c**), which indicates that long-chain aldehydes are less well accepted by the ω-TA, in addition to possible solubility issues.

Various functional groups were introduced at the C-terminal end of 1-alkanols, encompassing halogen, amino, alkyne, thiol, and nitrile functionalities (entries 9–16). For the ω-halo-1-alcohols, good-to-excellent conversions were obtained [74 % for **9a** (entry 9) and nearly full conversion for the C_6_ and C_8_ chloro analogues **10a** and **11a** (entries 10, 11). The introduction of a terminal nitrile group possessing similar properties as a halogen group (i.e. a pseudohalogen) led to a significant decrease in conversion (**12 a**, conv. 42 %, entry 12), whereas a terminal ethynyl group of similar size resulted in full conversion (**13 a**, conv.>99 %, entry 13).

The limitations of the cascade are set by the incompatibility of amino, hydroxy (diol), and thiol substituents (**14 a**–**16 a**, entries 14–16). Steric factors can be neglected, therefore, the nonacceptance of substrates bearing polar end groups is most likely because of their heavy hydration in aqueous medium. The same is true for secondary aliphatic and activated benzylic or allylic alcohols, which were not converted independent of their *E*/*Z* configuration (Figure S5).

In view of the preparative-scale application of the oxidation–amination cascade, a seven-fold upscale of the optimized screening conditions was performed with hydroxy-nitrile **12 a** and hydroxy-alkyne **13 a** as substrates. Both products were isolated after derivatization to the corresponding ethyl *N*-carbamates of **12 b** and **13 b** in 42 % and with complete conversion in case of the ω-nitrile analogue.

In conclusion, a five-enzyme cascade for the amination of primary alcohols by coupling a long-chain alcohol oxidase with a ω-transaminase has been successfully extended to also encompass nonactivated fatty alcohols as substrates. Long-chain alcohol oxidase from *A. fumigatus* was exploited as an excellent oxidation catalyst for a broad range of ω-functionalized aliphatic C_4_–C_11_ alcohols, which gave the corresponding primary amines with good-to-excellent conversion by using ω-TA from *C. violaceum*.

## Experimental Section

### General procedure for amination of alcohols

Lyophilized whole *E. coli* BL21(DE3) cell preparations containing overexpressed genes of ω-TA_Cv^18^ (20 mg) and LCAO_Af (40 mg) were both resuspended in sodium phosphate buffer (500 μL each, 100 mm, pH 10) supplemented with pyridoxal-5′-phosphate (PLP, 2 mm), NAD^+^ (2 mm) and FAD (1 mm). The samples were shaken at 30 °C and 120 rpm for 30 min in Eppendorf vials (horizontal position) and were combined after rehydration. A solution of l-alanine (100 mm), ammonium chloride (67 mm), and d-glucose (80 mm) in sodium phosphate buffer (500 μL, 100 mm, pH 10.0) was added. Alanine dehydrogenase (Ala-DH) from *B. subtilis* (0.013 U), glucose dehydrogenase (GDH from DSM, 2 U), and catalase (1700 U) from *M. lysodeikticus* were added. Finally, the substrate (10 mm) was added and the reaction mixture was placed into an oxygen pressure chamber.^19^ The apparatus was primed with oxygen (technical grade) for about 1 min and pressurized to 2 bar. The reaction mixture was shaken at RT and 170 rpm for 24 h. The conversion was determined by GC-MS analysis after derivatization of the corresponding primary amine with ethyl(succinimidooxy)formate (see the Supporting Information).

## Supporting Information

As a service to our authors and readers, this journal provides supporting information supplied by the authors. Such materials are peer reviewed and may be re-organized for online delivery, but are not copy-edited or typeset. Technical support issues arising from supporting information (other than missing files) should be addressed to the authors.
