# Supplementary material for: Amination of ω-Functionalized Aliphatic Primary Alcohols by a Biocatalytic Oxidation–Transamination Cascade
Source: ChemCatChem. 2015 Sep 3;7(19):3121–4. doi: 10.1002/cctc.201500589 (PMC4641459; doi:10.1002/cctc.201500589)
Supplement: Supplementary file 1 — Supplementary [file cctc0007-3121-sd1.pdf]

Heterogeneous & Homogeneous & Bio- & Nano-
CHEMCATCHEM
CATALYSIS

Supporting Information

Amination of ω-Functionalized Aliphatic Primary Alcohols by a Biocatalytic Oxidation–Transamination Cascade

Mathias Pickl,[b] Michael Fuchs,[b] Silvia M. Glueck,[a, b] and Kurt Faber*[b]

cctc_201500589_sm_miscellaneous_information.pdf

General information	2
Search for long chain alcohol oxidases	2
Cloning and overexpression of LCAO_Af	2
SDS-PAGE of LCAO_Af	4
Optimization studies	4
H_2O_2 cleavage	5
Cofactor recycling	5
pH profile	6
Substrate concentration	6
Ratio of LCAO_Af versus w-TA_Cv	7
Oxygen supply	7
Time study	8
Upscaling	9
Analytics	10
GC-MS method	10
General derivatization procedure	11
Synthesis of substrate **12a**	11
Non-substrates	11
NMRs of **12a**, **12b**$_{deriv.}$ and **13b**$_{deriv.}$	13
GC-MS of **1b-13b**	18
References	24

General information

All chemicals were purchased from Sigma Aldrich, except L-alanine (Alfa Aesar) and NAD^+ (BASF) and were used as received, solvents were obtained from Roth. Petroleum ether and ethyl acetate were distilled prior to use. Ethyl(succinimidooxy)formate was synthesized according to literature:[1] Rehydration of enzymes and biocatalytic reactions were performed in a HT Infors Unitron AJ 260 incubator with 120 rpm shaking (horizontal position) at 30°C or in an oxygen pressure chamber apparatus as previously described.[2] Centrifugation was done at 13000 rpm in a Heraeus Biofuge pico or at 4000 rpm in a Heraeus Biofuge primo. NMR-spectra were recorded on a Bruker NMR at 300 (^1H) and 75 (^{13}C) MHz using TMS as internal standard, shifts are given in ppm and coupling constants (*J*) are given in Hz. The activity of alanine dehydrogenase (measured by deamination of L-alanine to pyruvate) was 12.6 U/mL. For plasmid preparation and handling of ω-TAs see previously published procedures.[3] Formate dehydrogenase (FDH) was obtained from Evocatal (evo 1.1.230 – E130595.01, EC 1.2.1.2), horseradish peroxidase (HRP, # P8125, EC 1.11.1.7) and catalase from *Micrococcus lysodeikticus* (# 60634, EC 1.11.1.6) were purchased from Sigma Aldrich and glucose dehydrogenase (GDH 0.001 DSM) was obtained from DSM.

Search for long chain alcohol oxidases

A search for suitable alcohol oxidases was performed by screening the NCBI database for genes encoding putative enzymes. Redundant hits were eliminated and a set of selected genes were phylogenetically analyzed. Long chain alcohol oxidases from *Aspergillus fumigatus* (LCAO_Af) and from *Candida tropicalis* (LCAO_Ct) were cloned, overexpressed and tested for fatty alcohol oxidation. No activity was observed for LCAO_Ct and this ezyme was therefore not further investigated.

Cloning and overexpression of long chain alcohol oxidase from *Aspergillus fumigatus* (LCAO_Af)

Amino acid sequence of LCAO from *Aspergillus fumigatus* Af293 (accession number: XP_753079.1)[4]

MAEQAVTAYVPLDVPLPPIPEGQVFSDLQWRTLLSLADTVIPSIRSTSLPKSVSTKVVPESTF
KDAVSTLASHIHDPDATQIAEQYLEENASANPQFVEGLRRLFAEYIHEEGKSGINLILNALN
SKAGSLILTGSTTPIQDQPFEIREKIFSSWETSRIKPLRAIYRAFTAIFKKTWTVSPTIRSVVGC
PRVPIHGKPADGFEYEFLQFPPGAEPETIDTDVVIVGSGCGGSVAAKLAEAGYRVLVVEKS
YHYPSKYFPMDFNEGFVSMFENGGATTSDDGSIAVLAGSTWGGGGTVNWSASLQTQGYV
RREWASKGLPFFESHEYQQALDRVCDRMGVSNDHTEHNYSNRVLLDGARKLGYAAQPVP
QNTGGSNHYCGYCTMGCHSCGKKGPRETFLADAAKAGTTFIEGFRADKIRFKNTKGGRVA

CGVEGTWTSRDSYLGTAGPDRTTRKVIINASKVIVSCGTLHSPLLLLRSGLKNPQIGRNLYL
HPVVLSCAVFDEEIRPWEGSALTIVVNEFEDQDGQGHGVKIENVVMLPALYLPTFPWRDGL
DYKLWAAKLPRMSGFIALTKERDAGRVYPDPADGRVRIDYTVSAYDRKHIVEALIATAKI
AYISGAREFHTSNREMPPFIRPTEASDPNAPEGVTNQALQAWIAVLRRKNPVDPERTQYAS
AHQMGTCRMGSSPRTSVVDPECQVWGTQGLYVMDASVFPSASGVNPMVTNMAIADWAS
RKVVRSLEKANHGKTVLARL

Amino acid sequence of LCAO from *Candida tropicalis* (accession number: CAB75353.1)[5]

MASFLPDKVDYKNVDTLLLLCDGIIHETTVDEIRNVIDPNFPEDKYEEYVKTFTKPSQTPGF
RETIYDIINANTTDAIHSFIILTTALDSRILAPTLTNSLTPIREMSLPERERLLASWRDSPIAAKR
RLFRLVSSLTLSTFVRLASDLHLKAIHYPERDLREKAYESQEIDPFKYSFEEKPKFDGAESYL
PDIDVIIIGSGAGAGVVAHTLANDGYKTLVLEKGRYFSNLELNFNDKDGVQELYQGGGAL
TTTNQQMFILAGSTFGGGTTVNWSACLKTPFKVRKEWYVDYGVEFAADETYDMAQDYV
WKQMGASTEGITHSLANNVVIEGAKKLGYKSKPIDQNSGGHPHHPCGFCHLGCKYGIKQG
SVNNWFRDAAANGSKFMQQVRVLQIINKKGIASGILCEDVATGVKFTITGPKKYVVAAGA
LNTPIVLNNSGFKNKHIGKNLTLHPVSTVFGDFGKEVQADHFHKSIMTSLCYEVADLDGKG
HGCRIETILNAPFIQASLLPWRGSDEVRRDLLRYNNMVAMLLTTRDTTSGSVSADPKKPEA
LIVDYDVSKFDKNAILQAFLITSDMLHIEGAKRILSPQAWVPIFESSKPRDERSIDDKDYVEW
RAKAAKIPFDSYGSAYGSAHQMSTCRMSGKGPKYGAVDTDGRLFECSNVYVADASVLPT
ASGANPMITTMTFARNIALGLAEDLKSKAKL

The codon optimized genes coding for LCAO_Af and LCAO_Ct were synthesized at GeneArt AG (Regensburg, Germany) with corresponding restriction sites *Nde*I (*N*-terminus) and *Xho*I (*C*-terminus). The working plasmid (pMA) carrying the desired genes were digested with *Nde*I and *Xho*I, the products purified, ligated into pET21a(+) and transformed in *E. coli* one shot TOP10. A single colony was picked to inoculate 10 mL LB medium supplemented with 100 µg/mL ampicillin. After overnight growth at 30 °C, the plasmid was isolated.
Chemically competent *Escherichia coli* BL21 (DE3) cells were transformed with the obtained plasmid and heterologous overexpression was performed as follows: LB medium [330 mL containing Trypton (10 g/L), yeast extract (5 g/L) and NaCl (5 g/L)] supplemented with the appropriate antibiotic [ampicillin (100 µg/mL)] was inoculated with ONC (3 mL) and incubated at 30°C and 120 rpm until an OD_{600} of 0.6–0.8 was reached. Then IPTG (0.6 mM) was added for induction and the cells were left overnight at 20°C and 120 rpm. The cells were harvested by centrifugation (2 x 15 min, 5000 rpm, 4°C), washed with a minimum amount of phosphate buffer

(100 mM, pH 7) and re-centrifuged under the same conditions to obtain a cell pellet which was frozen in liquid nitrogen and lyophilized overnight. Freeze-dried cells were stored at +4°C.

Amino acid sequences, cloning and overexpression of ω-TAs and alanine dehydrogenase from *Bacillus subtilis* have been previously reported.[2,6]

SDS-PAGE of LCAO_Af

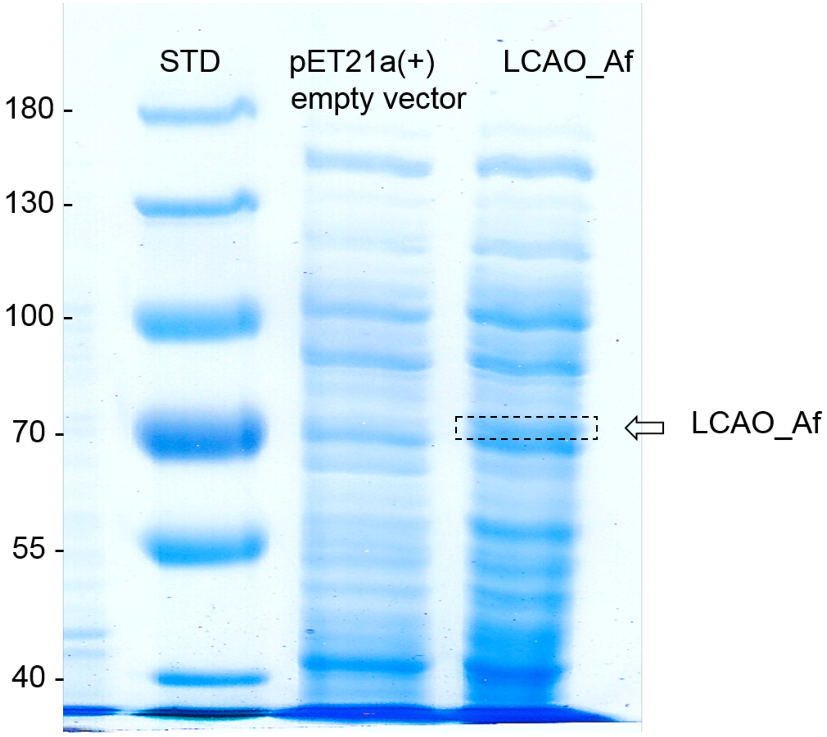

Figure S1. SDS-PAGE (7%); lane 1: protein standard (STD, PageRuler prestained protein ladder); lane 2: cell-free extract of pET21a(+) without LCAO_Af encoding gene (empty host); lane 3: cell-free extract of pET21a(+) with LCAO_Af encoding gene.

Optimization studies

1-Hexanol (**3a**) was used as model substrate for all optimization studies. LCAO_Af and ω-TA_Cv were applied as whole lyophilized *E. coli* cells containing the particular overexpressed oxidation or amination enzyme. The performance was determined as the overall conversion to the corresponding amine (1-hexylamine, **3b**), analyzed after derivatization to the corresponding ethyl *N*-carbamate.

Table S1. Evaluation of different methods for hydrogen peroxide cleavage.[a]

H_2O_2 cleavage system	product (3b) conv. [%]	
HRP/ABTS	47	±2
catalase	47	±2

[a] Reaction conditions: sodium phosphate buffer (100 mM, pH 7.0), L-alanine (100 mM), NAD^+ (2 mM), NH_4Cl (67 mM), PLP (2 mM), FAD (1 mM), LCAO_Af (20 mg lyophilized whole cells), ω-TA_Cv (20 mg lyophilized whole cells), GDH (2 U), D-glucose (80 mM), substrate **3a** (33 mM), Ala-DH (0.013 U), catalase (*M. lysodeikticus*, 1700 U) or HRP/ABTS (8 U/0.15 mg), incubation for 20 h at rt under 4 bar O_2 with 170 rpm shaking. Conversions were determined by GC-MS analysis after derivatization of **3b** to the corresponding ethyl *N*-carbamate.

Table S2. Evaluation of various NAD^+ recycling systems in the cascade system.[a]

cosubstrate	recycling enzyme	product (3b) conv. [%]	
D-glucose	GDH	75	±3
ammonium formate	FDH	90	±2
disodium hydrogen phosphite	PtDH	19	±7

[a] Reaction conditions: sodium phosphate buffer (100 mM, pH 7.0), L-alanine (100 mM), NAD^+ (2 mM), NH_4Cl (67 mM), PLP (2 mM), LCAO_Af (40 mg lyophilized whole cells), FAD (1 mM) ω-TA_Cv (20 mg lyophilized whole cells), catalase (*M. lysodeikticus*, 1700 U), substrate **3a** (33 mM), Ala-DH (0.013 U), incubation for 20 h at rt under 2 bar O_2 with shaking at 170 rpm. The following nicotinamide recycling systems were applied: GDH (2 U)/ D-glucose (80 mM), FDH (2 U)/ammonium formate (80mM) and PtDH (2 U)/disodium hydrogen phosphite (80mM). Conversions were determined by GC-MS analysis after derivatization of **3b** to the corresponding ethyl *N*-carbamate.

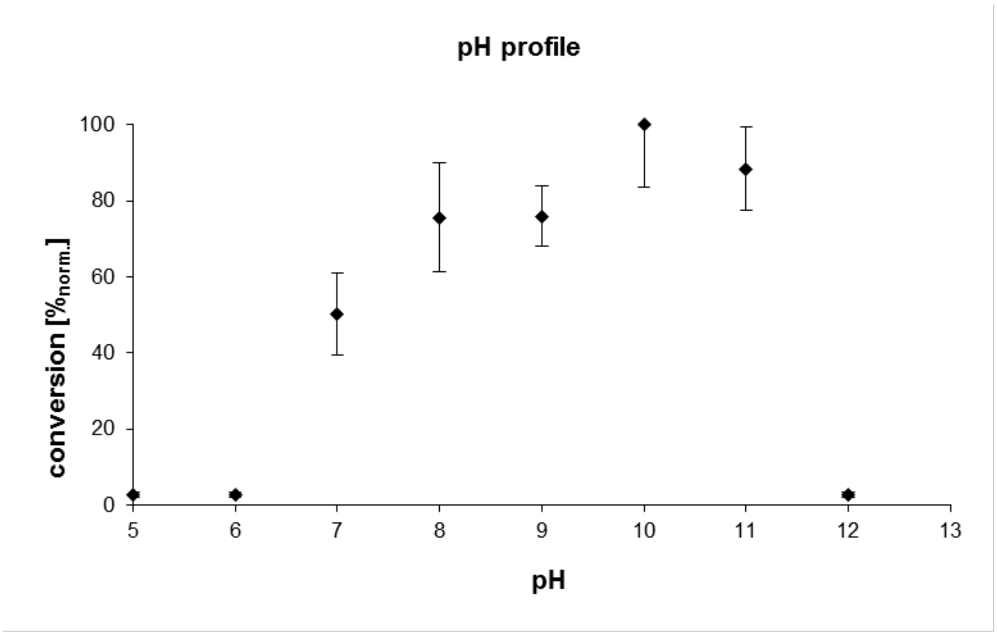

Figure S2. Evaluation of the pH profile of the cascade system. Reaction conditions: sodium phosphate buffer (100 mM, pH range from pH 5 - 12), L-alanine (100 mM), NAD^+ (2 mM), NH_4Cl (67 mM), PLP (2 mM), FAD (1 mM), LCAO_Af (20 mg whole lyophilized cells), ω-TA_Cv (20 mg whole lyophilized cells), GDH (2 U), D-glucose (80 mM), substrate **3a** (33 mM), Ala-DH (0.013 U), catalase (*M. lysodeikticus*, 1700 U), incubation for 20 h under 4 bar O_2 at rt with shaking at 170 rpm. Conversions were determined by GC-MS analysis after derivatization of **3b** to the corresponding ethyl *N*-carbamate and are normalized.

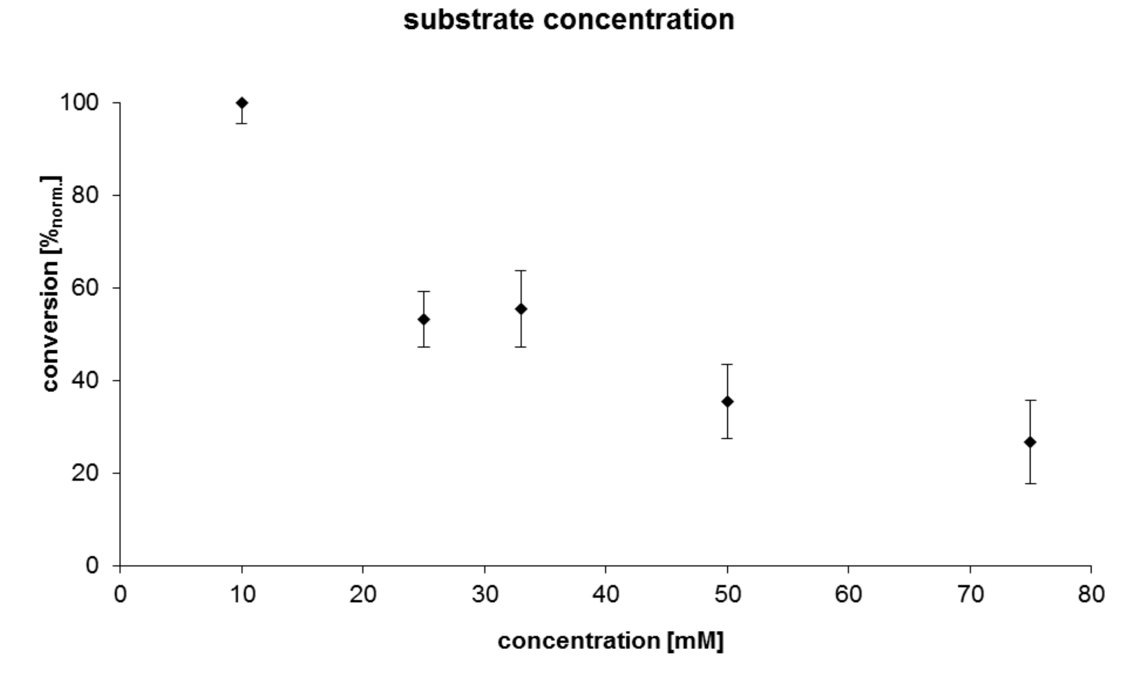

Figure S3. Performance of the amination cascade depending on the substrate concentration (**3a**). Reaction conditions: sodium phosphate buffer (100 mM, pH 7), L-alanine (100 mM), NAD^+ (2

mM), NH_4Cl (67 mM), PLP (2 mM), FAD (1 mM), LCAO_Af (20 mg whole lyophilized cells), ω-TA_Cv (20 mg whole lyophilized cells), GDH (2 U), D-glucose (80 mM), Ala-DH (0.013 U), catalase (*M. lysodeikticus*, 1700 U),various concentrations of substrate **3a** were applied (10 mM, 25 mM, 33 mM, 50 mM, 75 mM), incubation for 20 h under 4 bar O_2 at rt with shaking at 170 rpm. Conversions were determined by GC-MS analysis after derivatization of **3b** to the corresponding ethyl *N*-carbamate and are normalized.

Table S3. Ratio of long chain alcohol oxidase (LCAO_Af) vs. ω–transaminase (ω-TA_Cv)[a]

ratio LCAO_Af/ ω-TA_Cv	LCAO_Af [mg]	ω-TA_Cv [mg]	product (3b) conv. [$\%_{norm.}$]	
1:1	20	20	60	±2
2:1	40	20	67	±2
2.5:1	50	20	87	±3
3:1	60	20	>99	±1

[a] Reaction conditions: sodium phosphate buffer (100 mM, pH 7), substrate **3a** (33 mM), L-alanine (100 mM), NAD^+ (2 mM), NH_4Cl (67 mM), PLP (2 mM), FAD (1 mM), LCAO_Af (various amounts of whole lyophilized cells were applied: 20 mg, 40 mg, 50 mg and 60 mg); ω-TA_Cv (20 mg whole lyophilized cells), GDH (2 U), D-glucose (80 mM), Ala-DH (0.013 U), catalase (*M. lysodeikticus*, 1700 U), incubation for 20 h under 4 bar O_2 at rt with shaking at 170 rpm. Conversions were determined by GC-MS analysis after derivatization of **3b** to the corresponding ethyl *N*-carbamate and are normalized. The 2:1 ratio was used in further experiments due to handling issues of the cell suspension.

Table S4. Evaluation of oxygen pressure.[a]

pressure	product (3b) conv. [$\%_{norm.}$]	
atm (bench)	40	±5
1 bar O_2 (apparatus)	88	±1
2 bar O_2 (apparatus)	>99	±1
3 bar O_2 (apparatus)	96	±3
4 bar O_2 (apparatus)	>99	±1
5 bar O_2 (apparatus)	60	±4

[a] Reaction conditions: sodium phosphate buffer (100 mM, pH 7), substrate **3a** (33 mM), L-alanine

(100 mM), NAD$^+$ (2 mM), NH_4Cl (67 mM), PLP (2 mM), FAD (1 mM), LCAO_Af (20 mg whole lyophilized cells), ω-TA_Cv (20 mg whole lyophilized cells), GDH (2 U), D-glucose (80 mM), Ala-DH (0.013 U), catalase (*M. lysodeikticus*, 1700 U), various oxygen pressure was applied (atm, 1 – 5 bar), incubation for 20 h at rt with shaking at 170 rpm. Conversions were determined by GC-MS analysis after derivatization of **3b** to the corresponding ethyl *N*-carbamate and are normalized.

Time Study

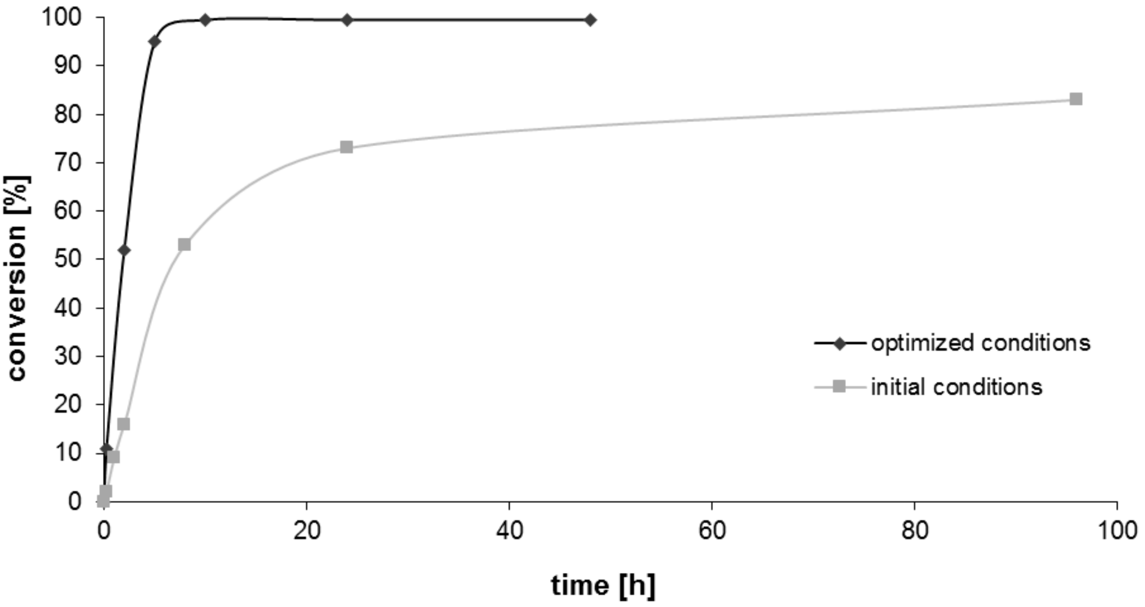

Figure S4. Performance of the amination cascade over time.

Initial reaction conditions: sodium phosphate buffer (100 mM, pH 7), L-alanine (100 mM), NAD$^+$ (2 mM), NH_4Cl (67 mM), PLP (2 mM), FAD (1 mM), LCAO_Af (20 mg whole lyophilized cells), ω-TA_Cv (20 mg whole lyophilized cells), GDH (2 U), D-glucose (80 mM), Ala-DH (0.013 U), catalase (*M. lysodeikticus*, 1700 U), substrate (33 mM), 4 bar O_2 at rt with shaking at 170 rpm. *Optimized reaction conditions:* sodium phosphate buffer (100 mM, pH 10), L-alanine (100 mM), NAD$^+$ (2 mM), NH_4Cl (67 mM), PLP (2 mM), FAD (1 mM), LCAO_Af (40 mg whole lyophilized cells), ω-TA_Cv (20 mg whole lyophilized cells), GDH (2 U), D-glucose (80 mM), Ala-DH (0.013 U), catalase (*M. lysodeikticus*, 1700 U), substrate (10 mM), 2 bar O_2 at rt with shaking at 170 rpm. Conversions were determined by GC-MS analysis after derivatization of **3b** to the corresponding ethyl *N*-carbamate.

Upscaling

Lyophilized whole *E. coli* BL21(DE3) cell preparations containing overexpressed genes of ω-transaminase from *C. violaceum* (ω-TA_Cv, 140 mg,) and long chain alcohol oxidase from *A. fumigatus* (LCAO_Af, 280 mg), respectively, were resuspended in sodium phosphate buffer (7 mL, 100 mM, pH 10.0) supplemented with PLP (2 mM), NAD^+ (2 mM) and FAD (1 mM).

The samples were shaken at 30 °C and 120 rpm for 30 min in a horizontal position and combined after rehydration. Afterwards a solution of L-alanine (100 mM), ammonium chloride (67 mM) and D-glucose (80 mM) in sodium phosphate buffer (3.5 mL, 100 mM, pH 10.0) was added. Alanine dehydrogenase from *B. subtilis* (70 µL, 0.09 U), glucose dehydrogenase (140 µL, 14 U) and catalase from *M. lysodeikticus* (70 µL, 11900 U) were added. Finally the substrate (10 mM) was added and the reaction mixture was placed into the oxygen apparatus. The apparatus was primed with oxygen (technical grade) for about 1 min and pressurized to 2 bar. The reaction mixture was shaken at rt and 170 rpm for 24 h (Rotilabo ® bottles with snap-on cap in vertical position).

Derivatization of amine products: ethyl(succinimidooxy)formate (210 mg, 1.05 mmol) dissolved in acetonitrile (3.5 mL) was added to the reaction mixture (10 mM substrate loading) followed by the addition of triethylamine (700 µL, 10 % v/v). The mixture was shaken at 45 °C and 700 rpm for 1 hour. Afterwards, the mixture was extracted twice with EtOAc (3.5 mL). The combined organic phases were dried over Na_2SO_4. The solvent was evaporated and the product was purified by flash chromatography (silica gel, petroleum ether/EtOAc 1:1).

NMRs of derivatized upscale-products (12b$_{\text{deriv.}}$, 13b$_{\text{deriv.}}$)

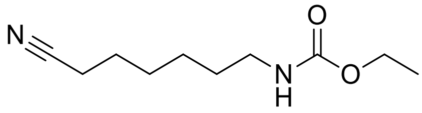

Ethyl *N*-(6-cyanohexyl)carbamate (**12b$_{\text{deriv.}}$**)

Yield: 1 mg, 15%

^{1}H NMR (300 MHz, $CDCl_3$): δ = 4.62 (s, 1H), 4.11 (q, 2H, *J* = 4.1 Hz), 3.16 (dd, 2H, *J* = 12.9 and 6.6 Hz, 2H), 2.35 (t, 2H, *J* = 7.0 Hz), 1.70-1.57 (m, 2H), 1.52 – 1.22 (m, 9H); ^{13}C NMR (75 MHz, $CDCl_3$): δ = 156.8, 119.8, 60.9, 40.8, 29.7, 28.5, 26.1, 25.4, 17.3, 14.8; GC-MS (70 eV): m/z = [%] 182 (1) [$M-CH_3^+$], 102 (100) [$C_4H_8NO_2^+$].

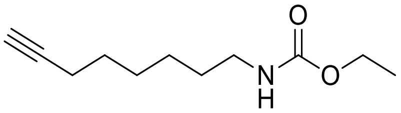

Ethyl *N*-oct-7-yn-1-ylcarbamate (**13b$_{\text{deriv.}}$**)

Yield: 5 mg, 30%

^{1}H-NMR (300 MHz, $CDCl_3$) δ = 4.62 (s, 1H), 4.12 (q, 2H, J = 4.1 Hz), 3.16 (dd, 2H, J = 13.1 and 6.6 Hz), 2.22-2.16 (m, 2H), 1.94 (t, 1H, J = 2.6 Hz,), 1.53 – 1.21 (m, 11H); ^{13}C-NMR (75 MHz, $CDCl_3$) δ = 156.8, 84.7, 68.4, 60.8, 41.0, 30.1, 29.8, 28.4, 26.4, 18.5, 14.8; GC-MS (70 eV): m/z = [%] 198 (1) [M^+], 102 (100) [$C_4H_8NO_2^+$].

Analytics

GC-MS method

Conversions were determined by GC-MS analysis on an Agilent 7890A GC system, equipped with an Agilent 5975C mass selective detector (EI 70 eV) and a HP-5-MS column (30 m x 0.25 mm, 0.25 µm film) using He at a flow rate of 0.5 mL/min. Temperature program: 40°C for 2 min, 20°C/min to 180°C for 1 min, 20°C/min to 300°C for 4 min. Run time was 20 min. Inlet temperature was 250°C.

Table S5: Retention times of alcohols (**1a-13a**), aldehyde intermediates, derivatized *prim*-amine products (**1b**$_{deriv.}$-**13b**$_{deriv.}$) and the corresponding carboxylic acids (**3c, 5c**-**8c**).

	**t_R [min]**			
**compound**	**alcohol (1a-13a)**	**aldehyde**	**derivatized amine (1b$_{deriv.}$-13b$_{deriv.}$)**	**carboxylic acid (3c, 5c-8c)**
**1**	3.54	n. d.	8.18	n. d.
**2**	4.51	n. d.	8.78	n. d.
**3**	5.74	4.90	9.61	6.80
**4**	6.69	n. d.	10.36	n. d.
**5**	7.90	n. d.	11.16	8.57
**6**	8.43	n. d.	11.93	9.07
**7**	9.37	n. d.	12.81	9.99
**8**	10.19	n. d.	13.46	10.80
**9**	8.18	n. d.	11.78	n. d.
**10**	9.79	n. d.	13.19	n. d.
**11**	8.85	n. d.	12.43	n. d.
**12**	9.34	n. d.	12.85	n. d.
**13**	7.96	n. d.	11.54	n. d.

n.d. = not determined, below the limit of detectability.

General derivatization procedure

For derivatization, ethyl(succinimidooxy)formate (30 mg, 0.15 mmol) dissolved in acetonitrile (500 µL) was added to the reaction mixture (10 mM substrate loading) followed by the addition of triethylamine (100 µL, 10 % v/v). The mixture was shaken at 45 °C and 500 rpm for 1 hour. Afterwards, the mixture was extracted twice with EtOAc (500 µL). The combined organic phases were dried over Na_2SO_4.

Synthesis of substrate 12a

7-Hydroxy-heptanenitrile (**12a**) was synthesized according to lit.[7] with following modifications: Powdered KCN (995 mg, 15.29 mmol, 2.0 eq) was dried under vacuum at 100°C for 8 h, then stored under argon overnight. After cooling to rt, dry CH_3CN (3 mL), 6-bromo-1-hexanol (1 mL, 7.643 mmol, 1.0 eq) and 18-crown-6 (202 mg, 0.7463 mmol, 0.1 eq) were added. The suspension was stirred for 30 h at 80°C. After cooling to rt, CH_3CN was removed by rotary evaporation and the resulting slurry was partitioned between dichloromethane (2 x 50 mL) and sat. Na_2CO_3 (50 mL). The organic phase was dried over Na_2SO_4 and evaporated. Flash-column chromatography (1:2 petroleum ether/EtOAc) provided 7-hydroxy-heptanenitrile (**12a**) as a colorless oil (793 mg, 82%); ^{1}H NMR (300 MHz, $CDCl_3$): δ = 3.65 (t, 2H, *J* = 7.0 Hz), 2.36 (t, 2H, *J* = 7.0 Hz), 1.71-1.38 (m, 8H); ^{13}C NMR (75 MHz, $CDCl_3$): δ = 119.8, 62.6, 32.3, 28.4, 25.3, 25.0, 17.1; GC-MS (70 eV): m/z = 126 [M-1].

After derivatization with ethyl(succinimidooxy)formate, the corresponding ethyl *N*-carbamate was obtained: ^{1}H-NMR (300 MHz, $CDCl_3$) *δ* = 4.39 (q, 2H, *J* = 7.1 Hz), 2.83 (s, 4H), 1.39 (t, 3H, *J* = 7.0 Hz); ^{13}C-NMR (300 MHz, $CDCl_3$) *δ* = 168.4, 151.2, 67.3, 25.2, 13.7; GC-MS (70 eV): m/z [%] = 187 [M^+] (1), 115 [$C_4H_5NO_3^+$] (100).

Non-substrates

The compounds shown in Figure S5 were not accepted by the enzymatic oxidation-transamination cascade.

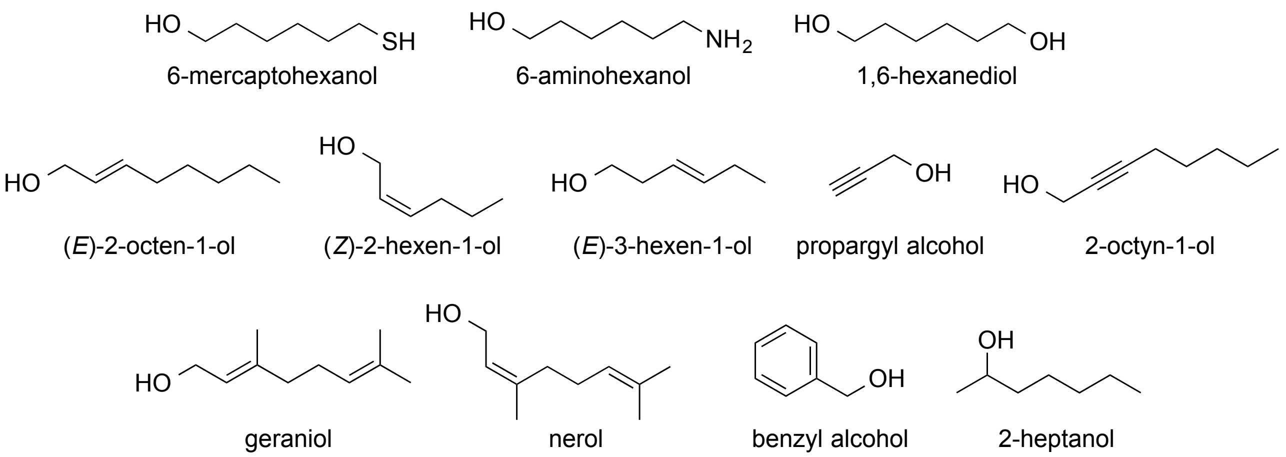

Figure S5: Non-substrates for the oxidation-amination cascade.

NMRs

7-Hydroxy-heptanenitrile (12a)

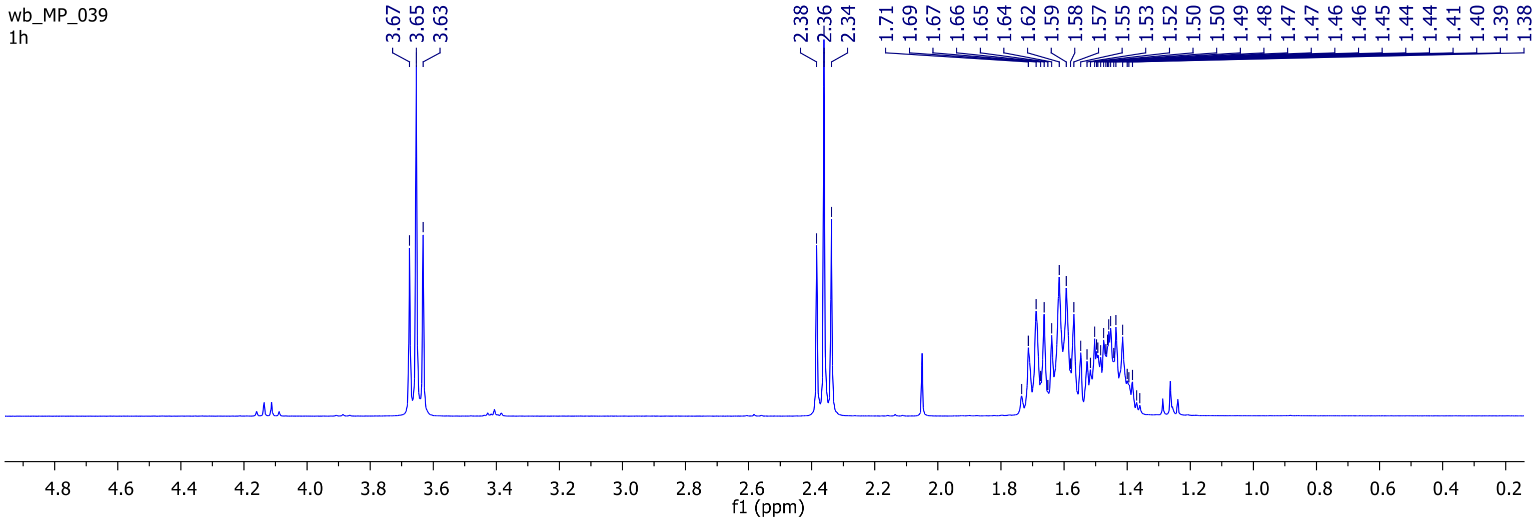

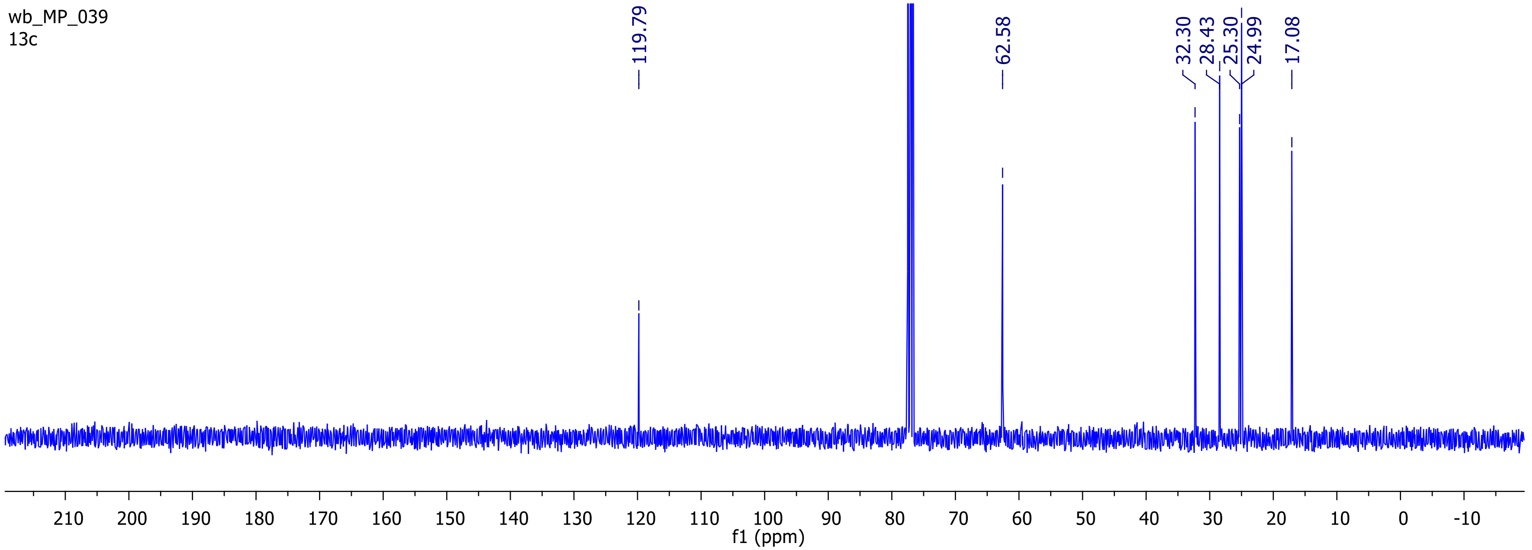

Ethyl *N*-(6-cyanohexyl)carbamate ($12b_{deriv.}$)

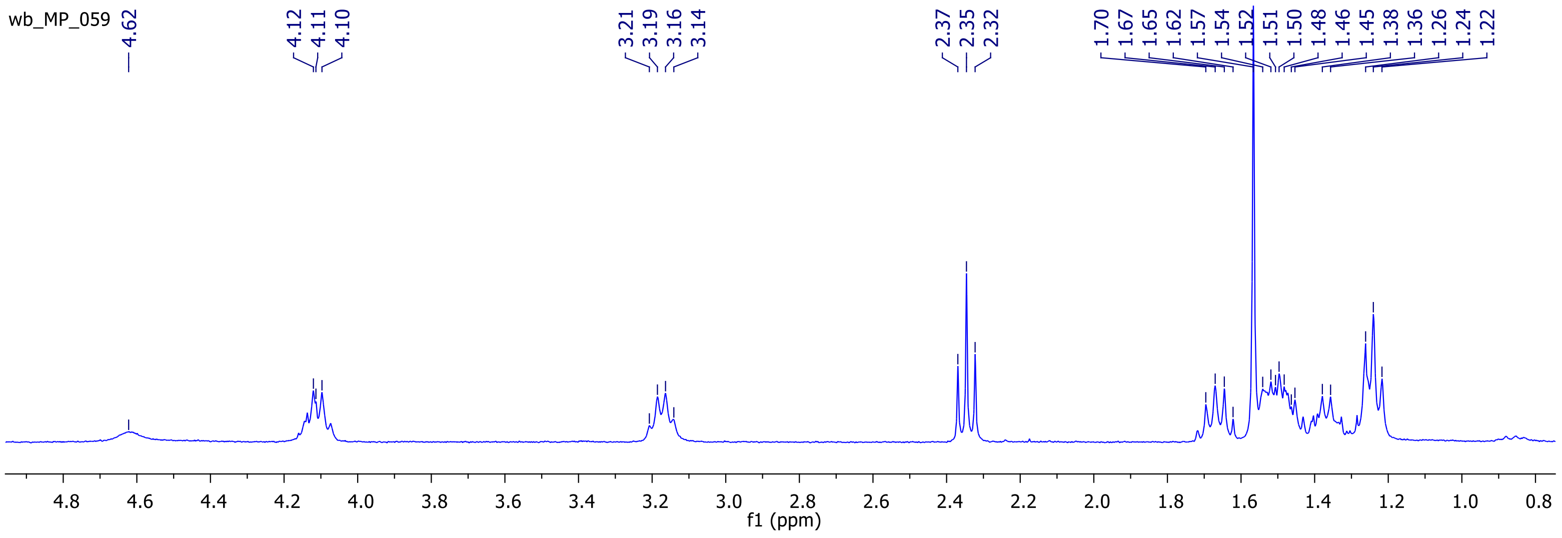

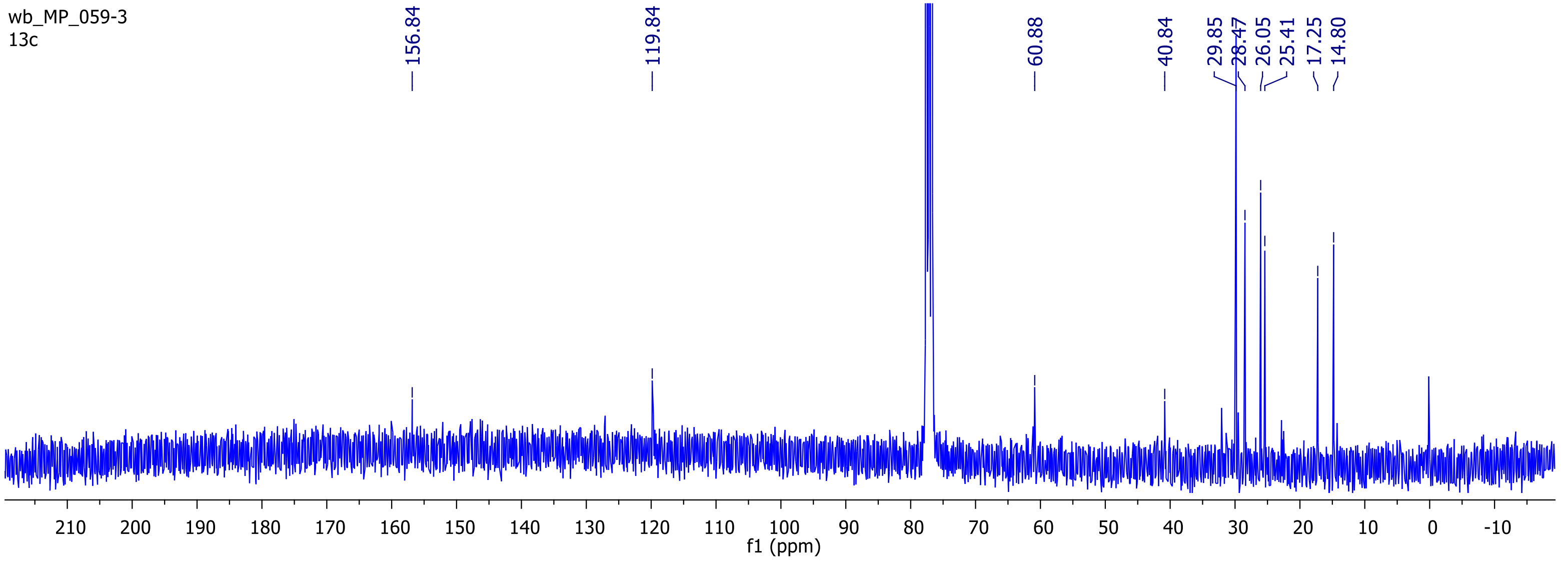

wb_MP_059-3

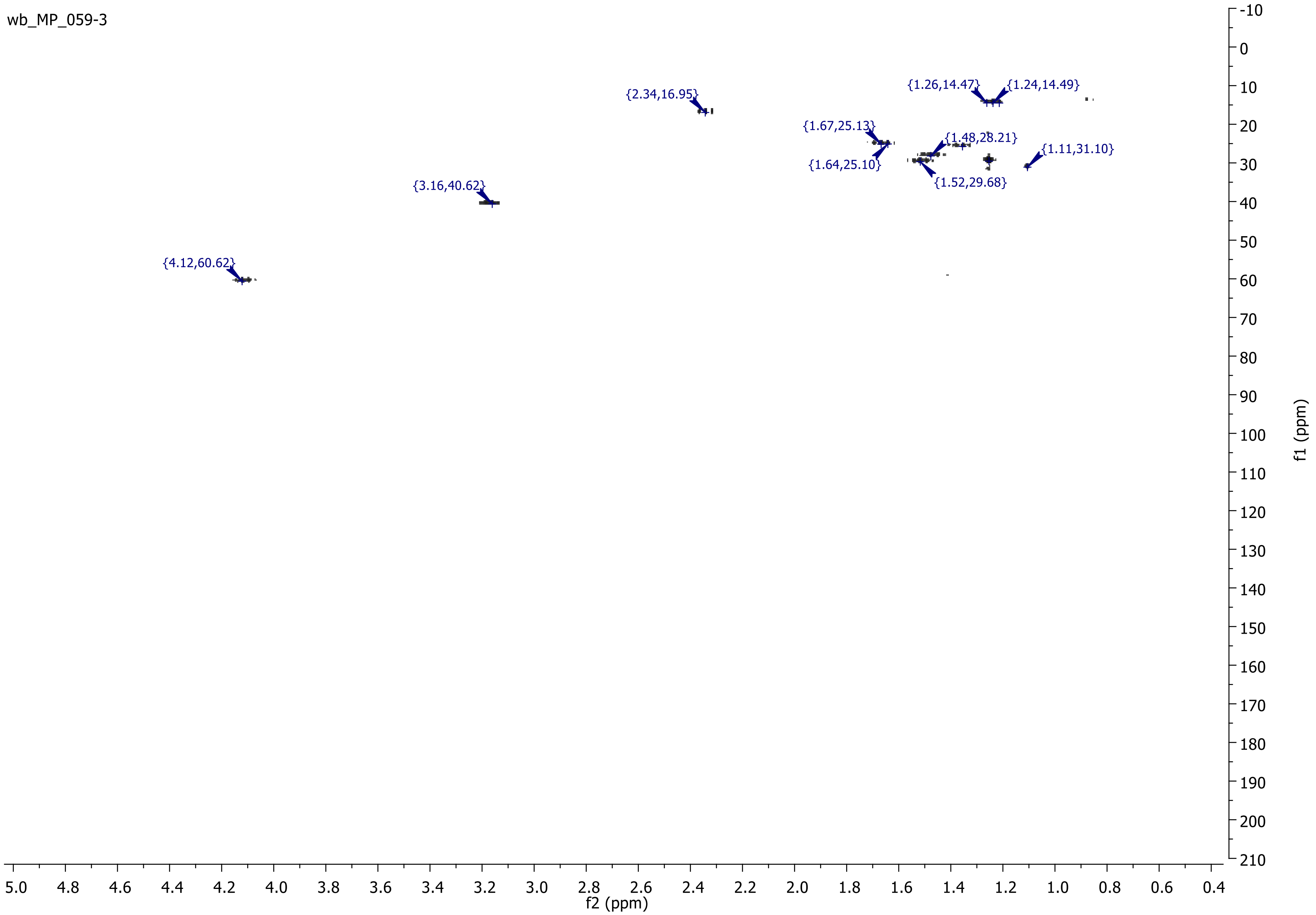
{2.34,16.95}
{1.26,14.47}
{1.24,14.49}
{1.67,25.13}
{1.48,28.21}
{1.11,31.10}
{1.64,25.10}
{1.52,29.68}
{3.16,40.62}
{4.12,60.62}
f2 (ppm)
f1 (ppm)

Ethyl *N*-oct-7-yn-1-ylcarbamate (13b$_{deriv.}$)

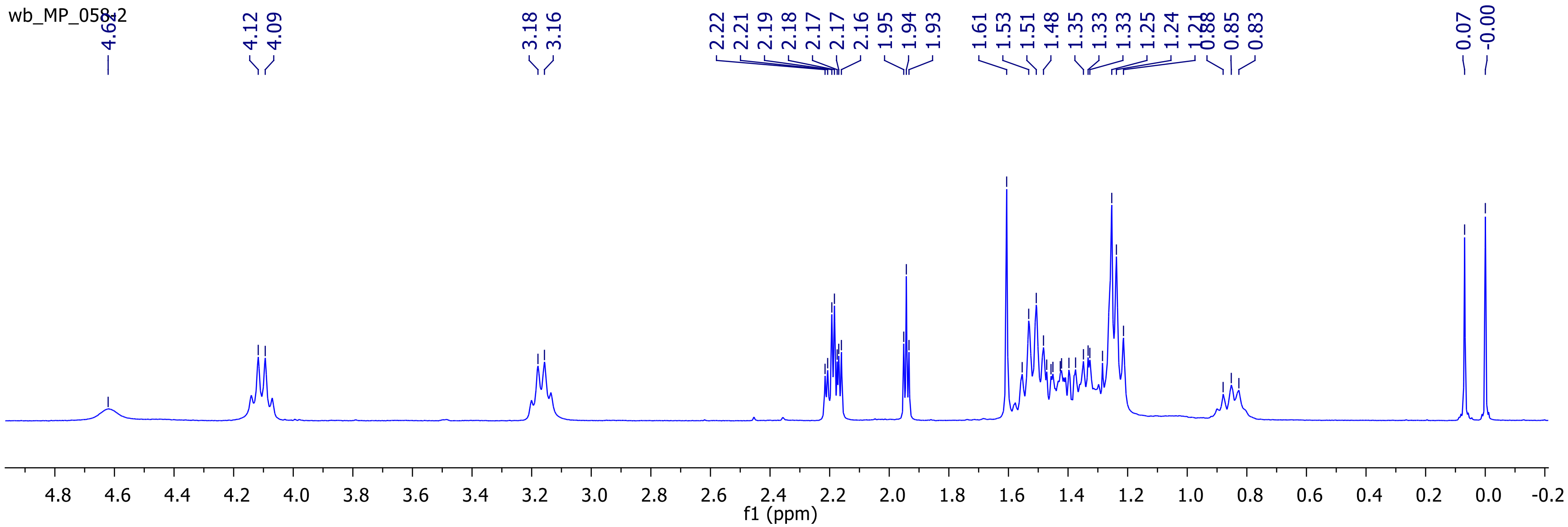

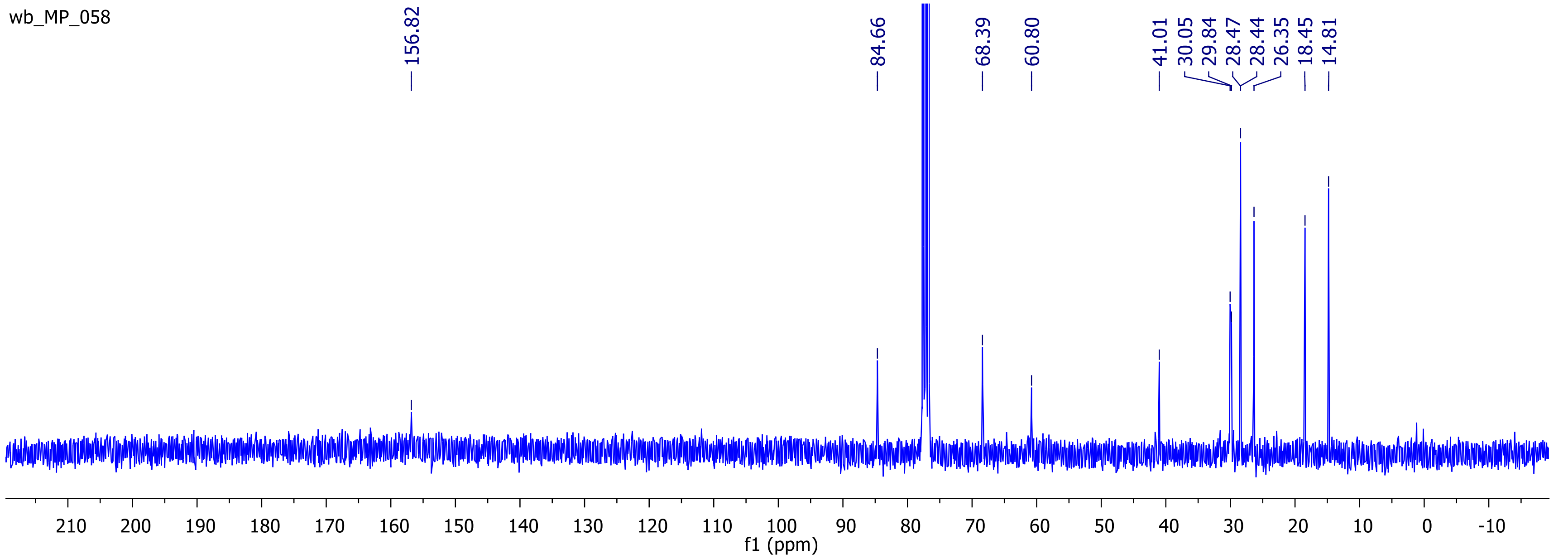

wb_MP_058-2
hsqc

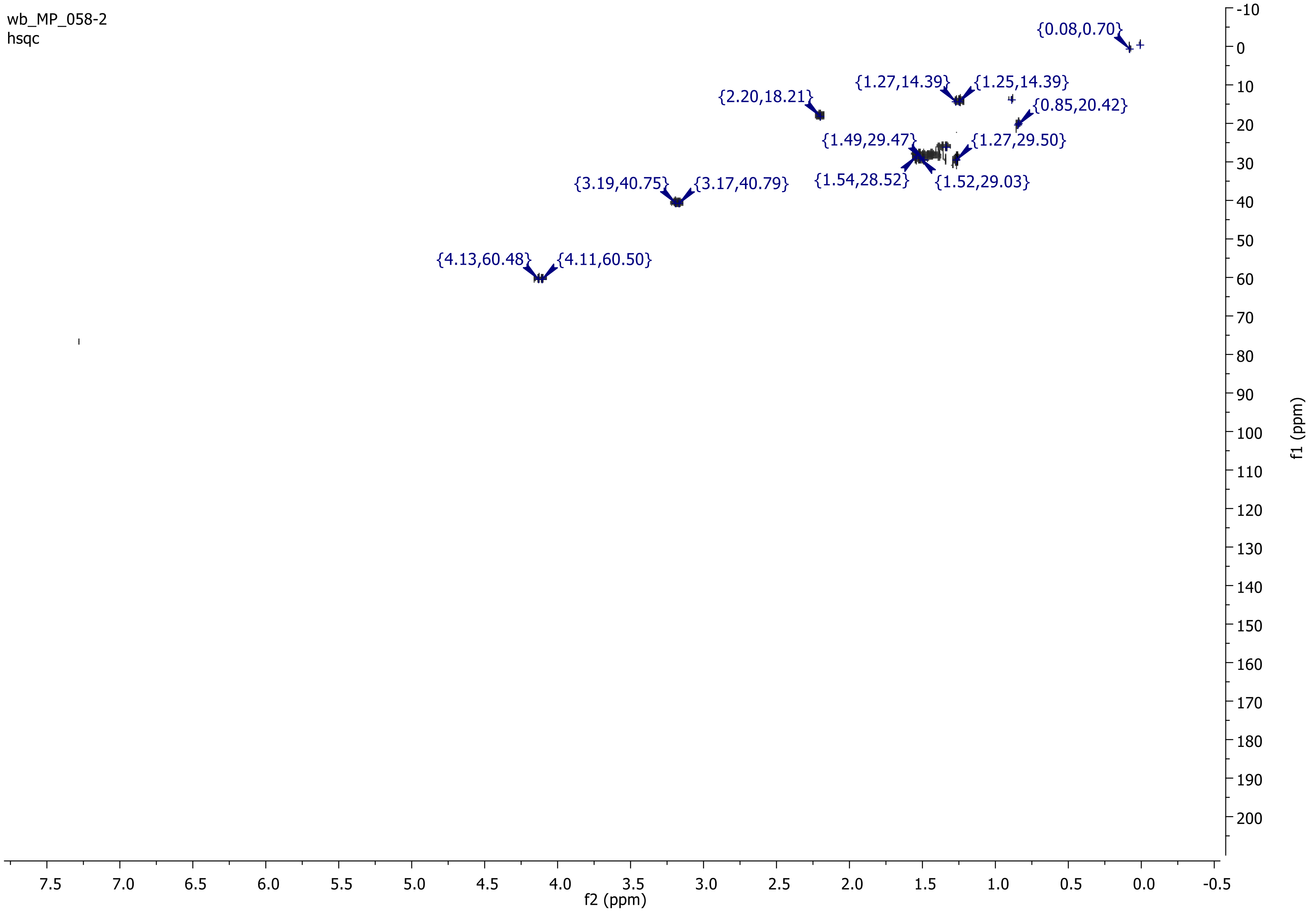

GC-MS of amines 1b$_{deriv.}$-13b$_{deriv.}$ derivatized as ethyl *N*-carbamates

1b$_{deriv.}$

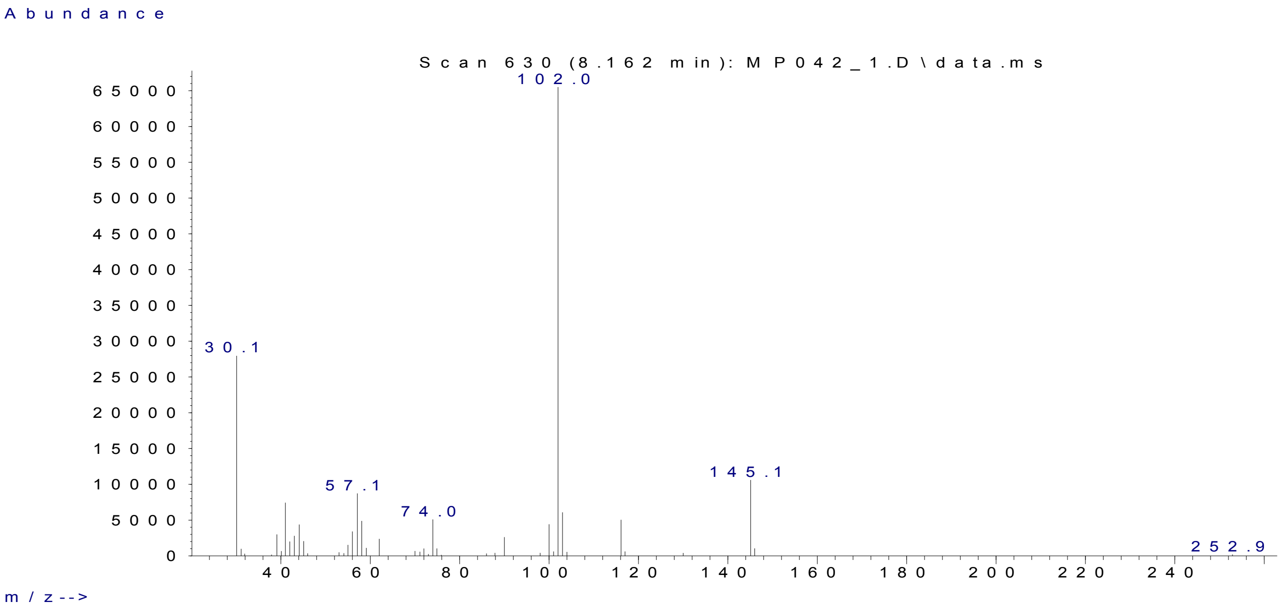

2b$_{deriv.}$

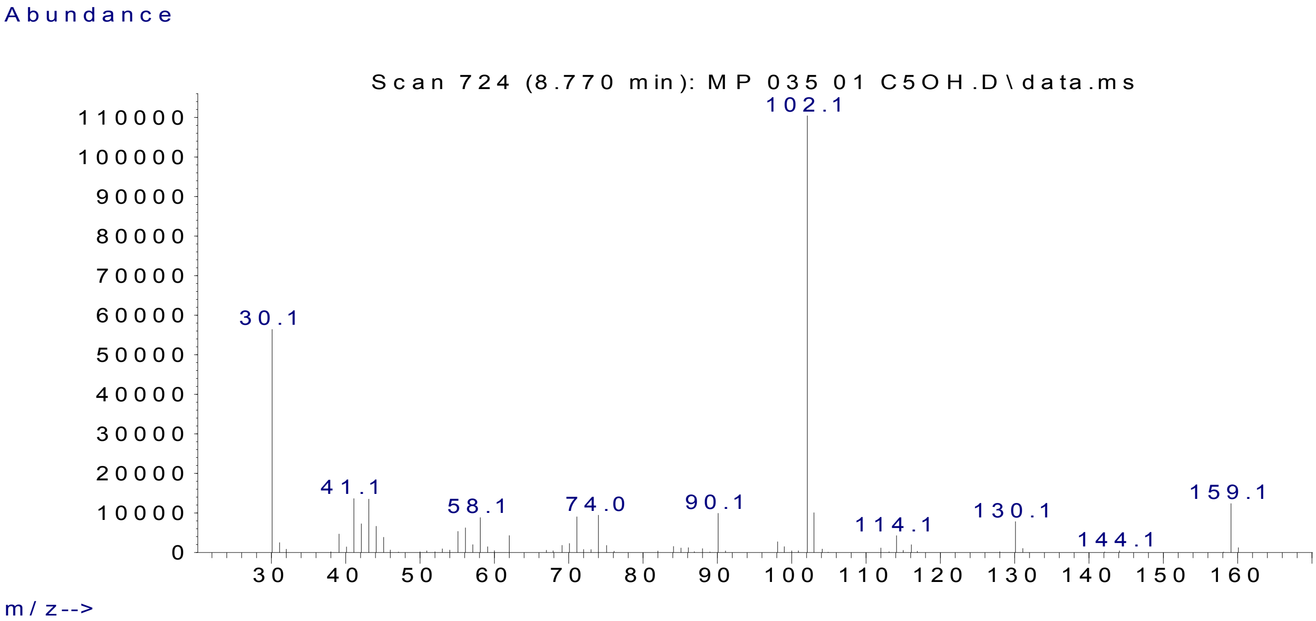

3bderiv.

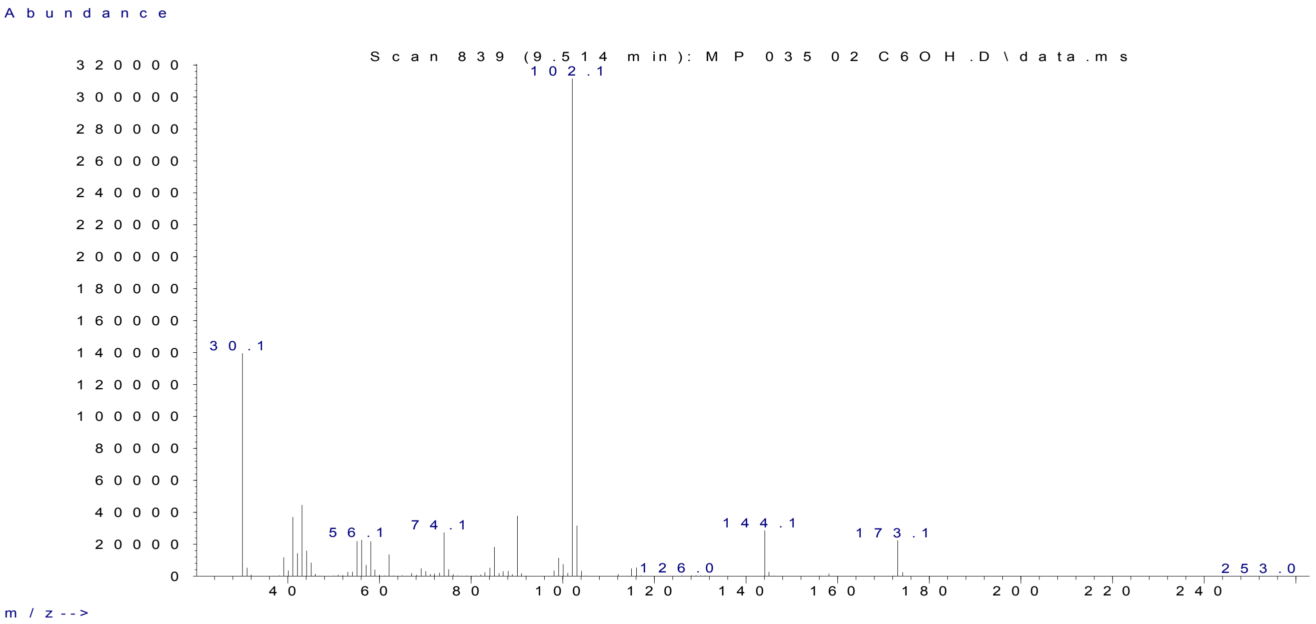

4bderiv.

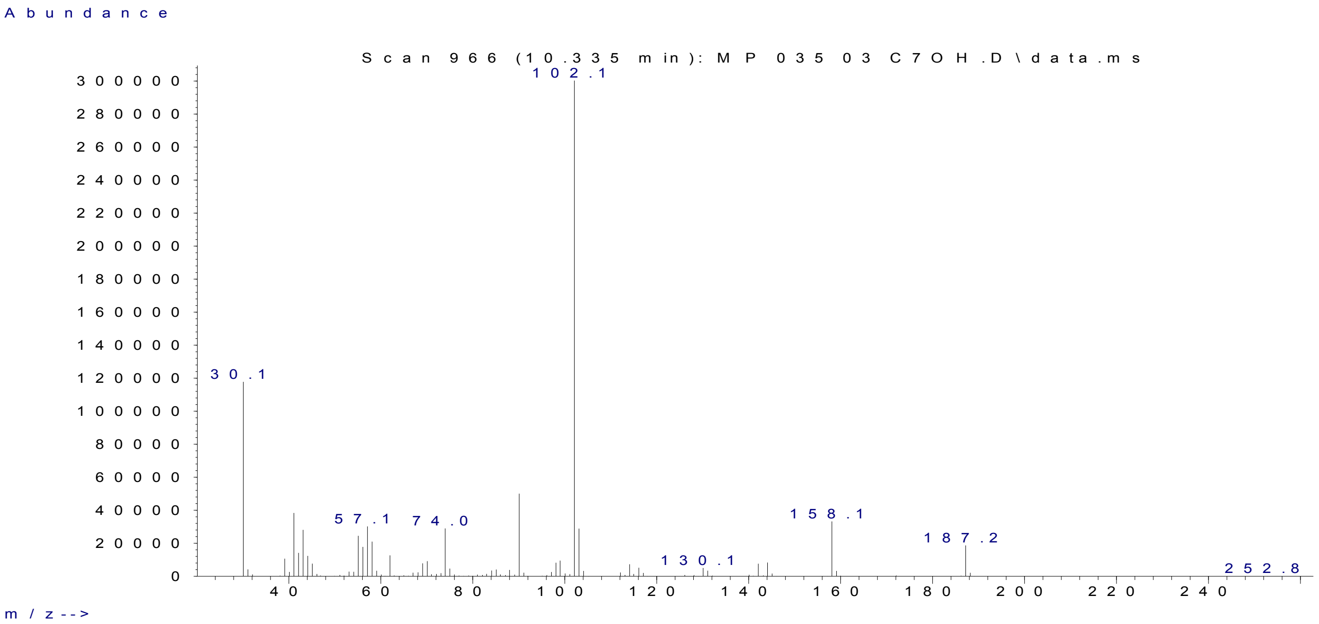

5bderiv.

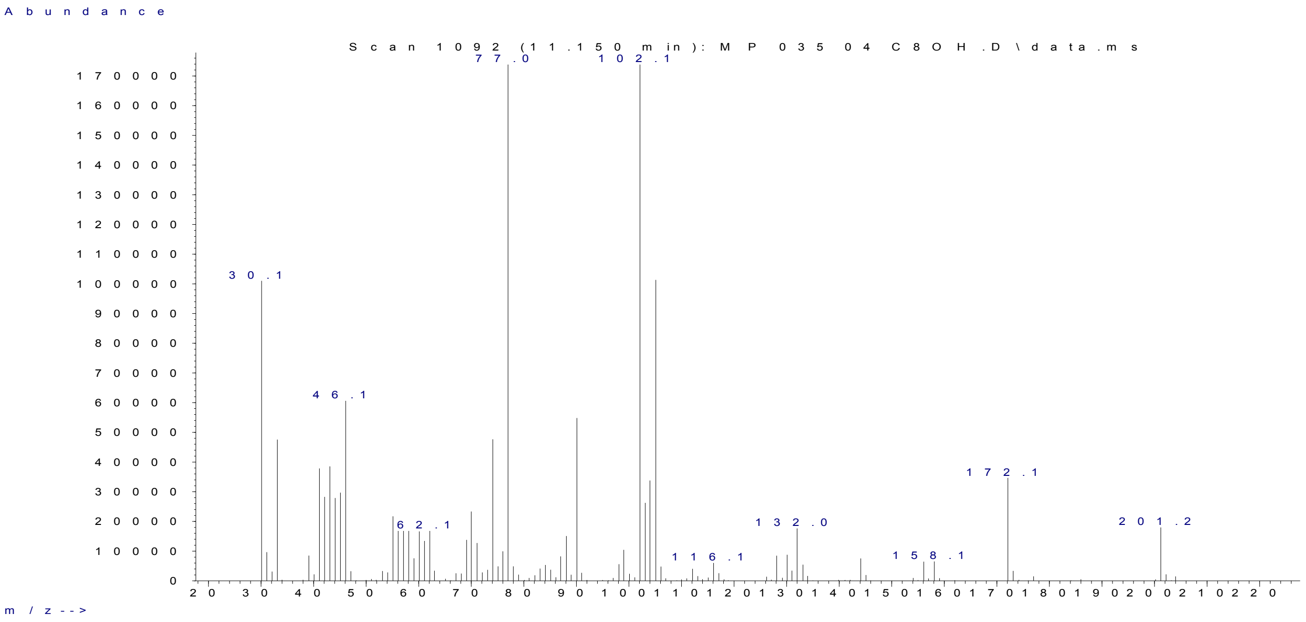

6bderiv.

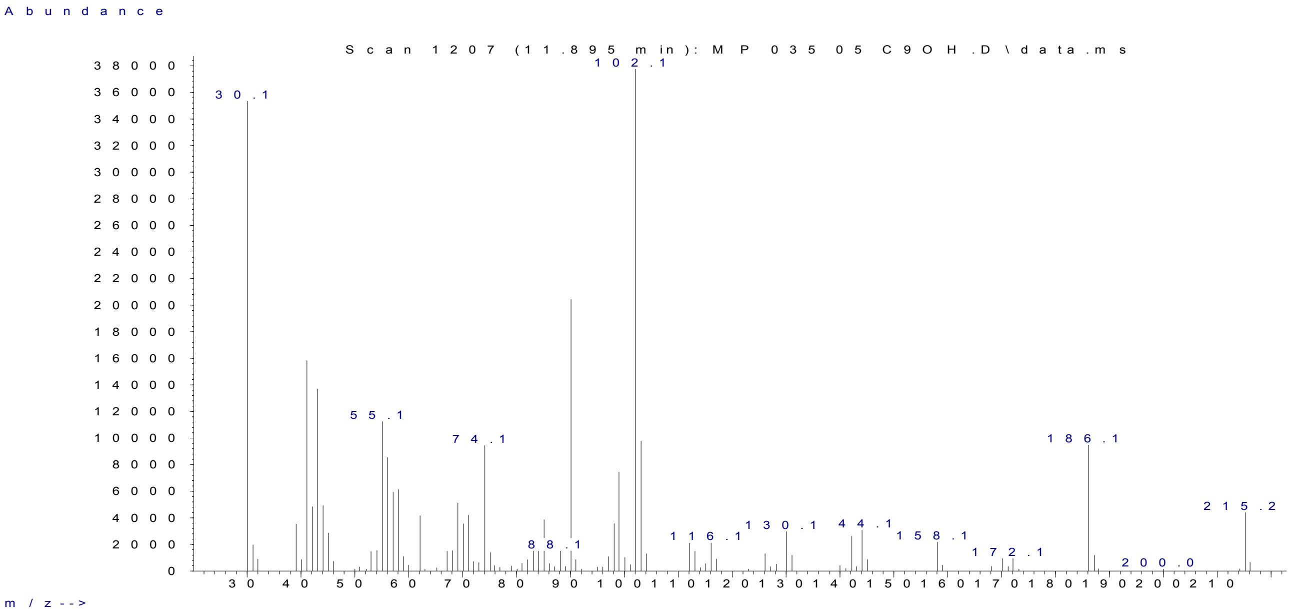

7bderiv.

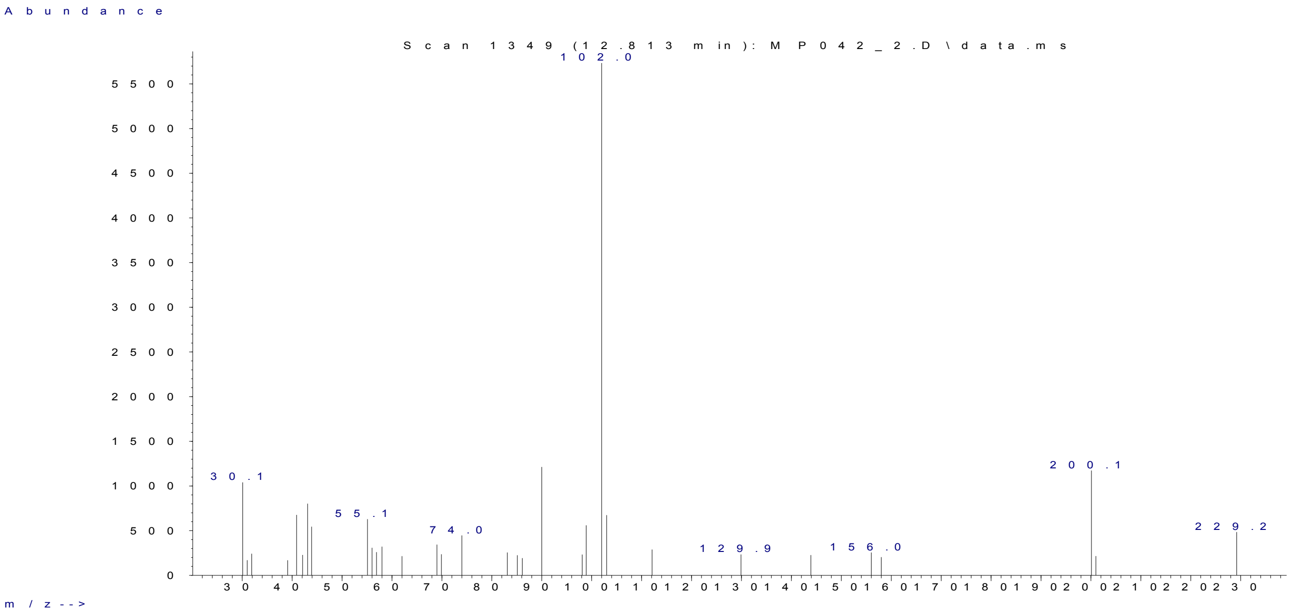

8bderiv.

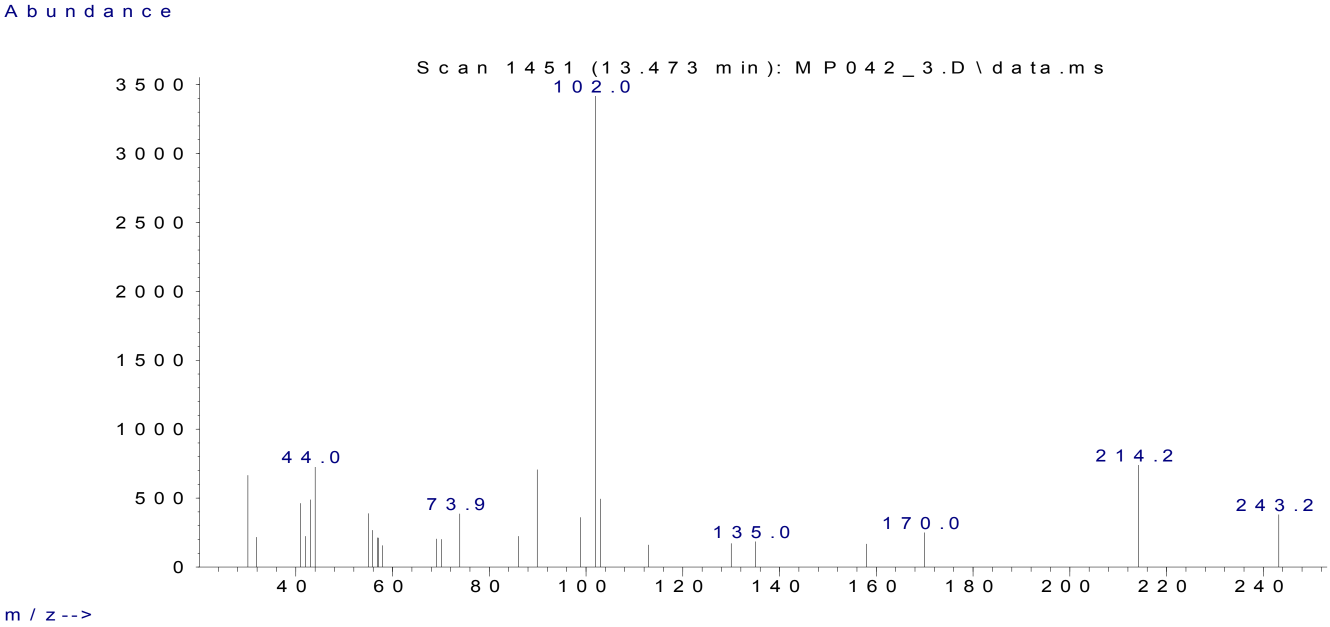

9bderiv.

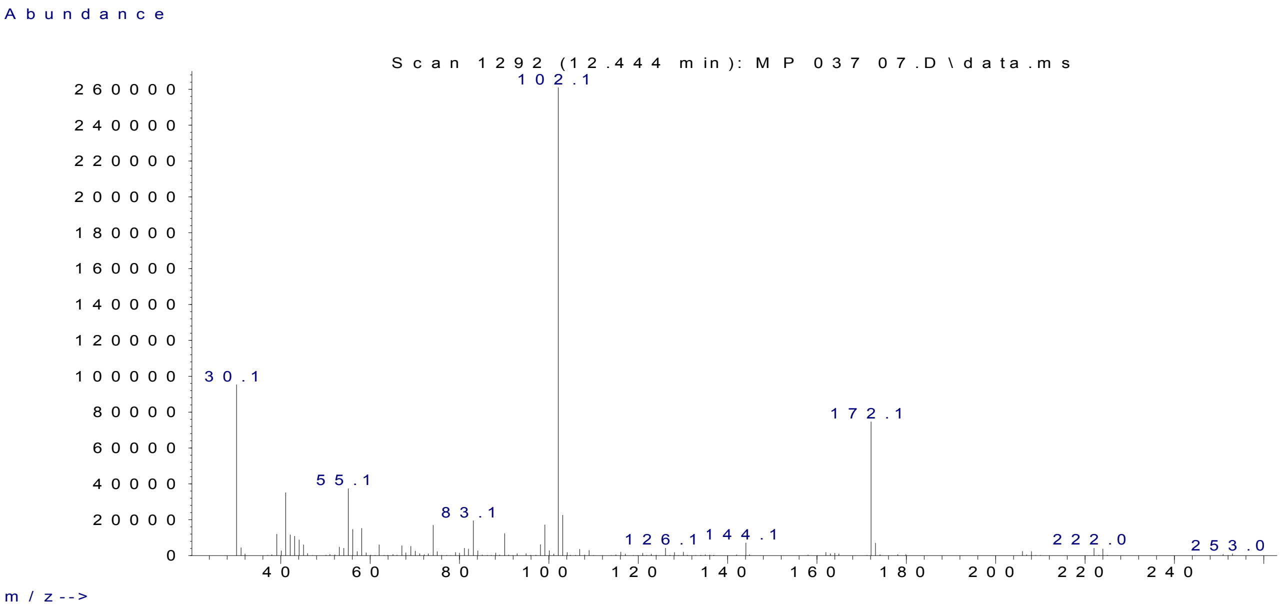

10bderiv.

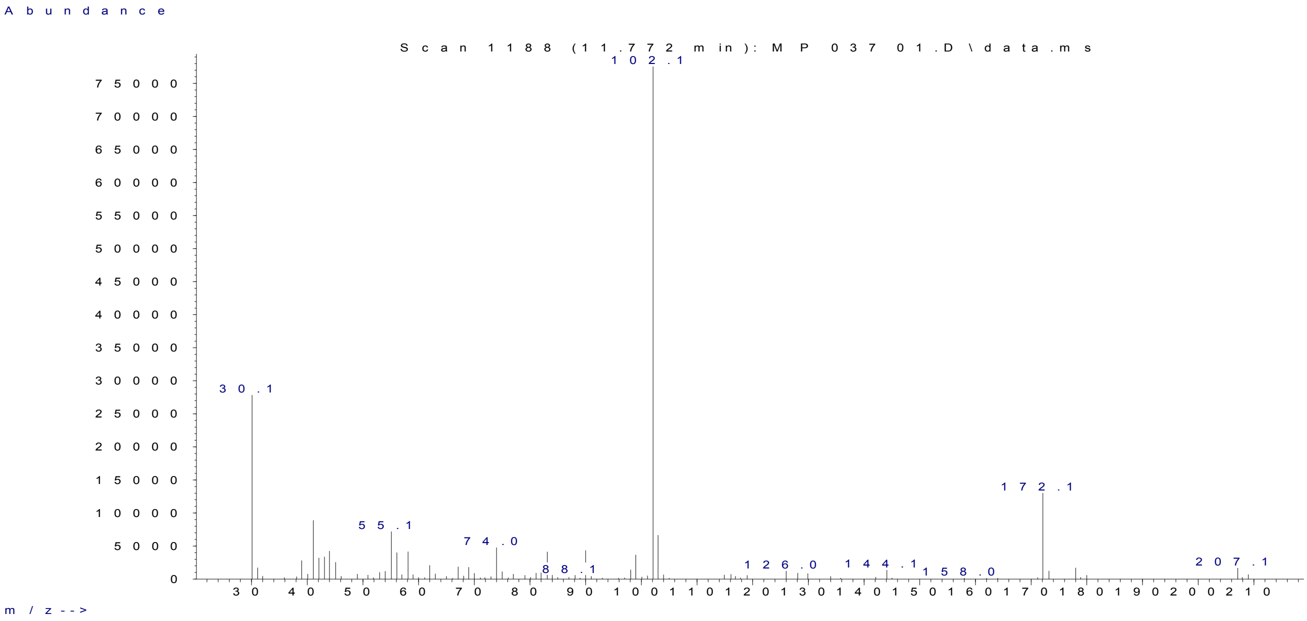

11bderiv.

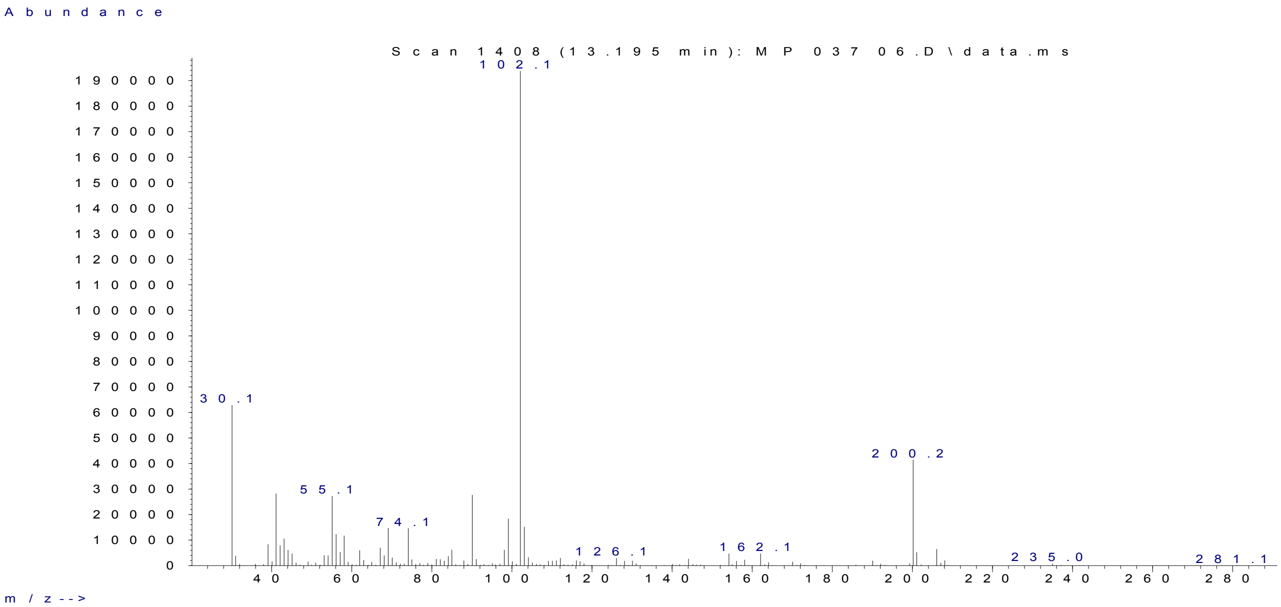

12bderiv.

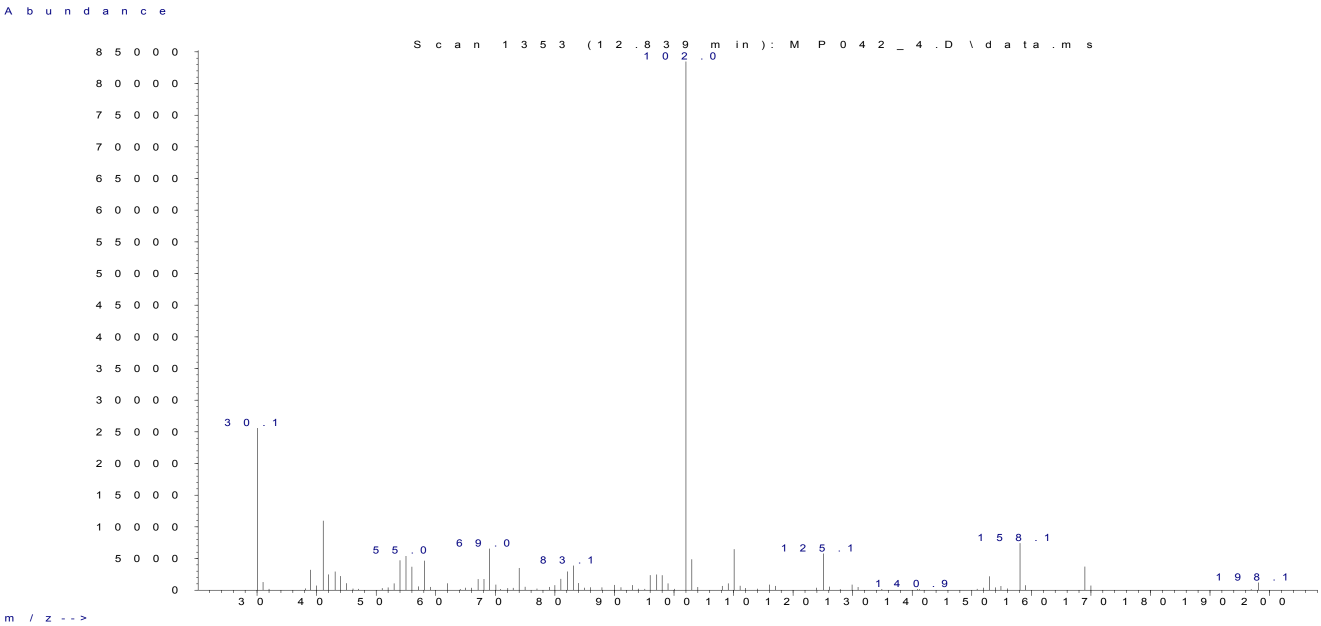

13b$_{deriv}$

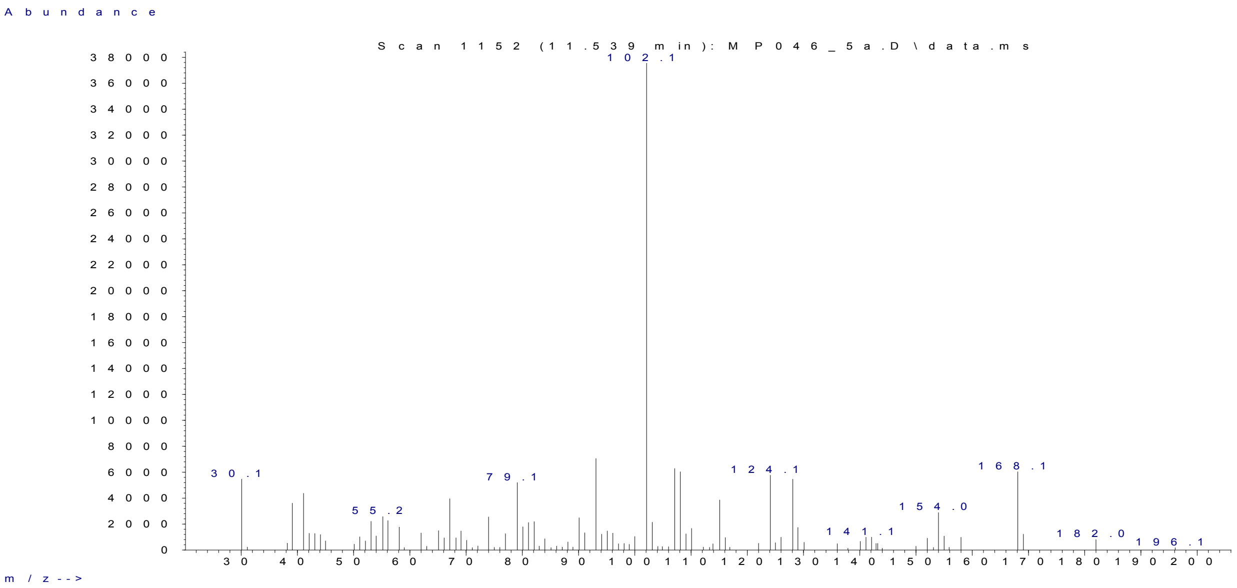

References

[1] I. Edafiogho, K. R. Scott, J. A. Moore, V. A. Famar, J. M. Nicholson, *J. Med. Chem.* **1991**, *34*, 387–392.

[2] M. Fuchs, M. Schober, J. Pfeffer, W. Kroutil, R. Birner-Gruenberger, K. Faber, *Adv. Synth. Catal.* **2011**, *353*, 2354–2358.

[3] D. Koszelewski, M. Goritzer, D. Clay, B. Seisser, W. Kroutil, *ChemCatChem* **2010**, *2*, 73–77.

[4] W. C. Nierman, A. Pain, M. J. Anderson, J. R. Wortman, H. S. Kim, J. Arroyo, M. Berriman, K. Abe, D. B. Archer, C. Bermejo, et al., *Nature* **2005**, *438*, 1151–1156.

[5] S. Vanhanen, M. West, J. T. M. Kroon, N. Lindner, J. Casey, Q. Cheng, K. M. Elborough, A. R. Slabas *J. Biol. Chem.* **2000**, *275*, 4445-4452.

[6] M. Fuchs, K. Tauber, J. Sattler, H. Lechner, J. Pfeffer, W. Kroutil, K. Faber, *RSC Adv.* **2012**, *2*, 6262–6265.

[7] D. C. Johnson II, T. S. Widlanski, *J. Org. Chem.* **2003**, *68*, 5300–5309.
